# Live auxotrophic vaccines contribute to protection against chronic lung infections caused by *Pseudomonas aeruginosa* in C57BL/6 mice

**DOI:** 10.3389/fimmu.2026.1862061

**Published:** 2026-09-03

**Authors:** Víctor Fuentes-Valverde, Alexandra Correia, Patricia García, Elena Pérez-Antón, Irina Amorim, Soraya Rumbo-Feal, Maria P. Cabral, Manuel Vilanova, Miriam Moscoso, Germán Bou

**Affiliations:** 1 Servicio de Microbiología Clínica and Grupo de Investigación en Microbiología, Instituto de Investigación Biomédica de A Coruña (INIBIC), Complexo Hospitalario Universitario de A Coruña (CHUAC), Servizo Galego de Saúde (SERGAS), Universidade da Coruña, A Coruña, Spain; 2 Centro de Investigación Biomédica en Red de Enfermedades Infecciosas (CIBERINFEC), Instituto de Salud Carlos III, Madrid, Spain; 3 i3S Instituto de Investigação e Inovação em Saúde, Universidade do Porto, Porto, Portugal; 4 Departamento de Imuno-Fisiologia e Farmacologia, Instituto de Ciências Biomédicas Abel Salazar, Universidade do Porto, Porto, Portugal; 5 Departamento de Patologia e Imunologia Molecular, Instituto de Ciências Biomédicas Abel Salazar, Universidade do Porto, Porto, Portugal; 6 Departamento de Fisioterapia, Medicina e Ciencias Biomédicas, Universidade da Coruña, A Coruña, Spain

**Keywords:** auxotrophy, chronic lung infection, IL-17, live vaccines, protective efficacy, *Pseudomonas aeruginosa*

## Abstract

*Pseudomonas aeruginosa* causes severe, often multidrug-resistant lung infections, especially in individuals with cystic fibrosis, highlighting vaccination as a promising preventive approach. This study evaluated intranasal and intradermal immunization with a D-glutamate auxotrophic *P. aeruginosa* strain (PAO1 Δ*murI*) and two of its derivatives, one with additional D-alanine auxotrophy and another exhibiting reduced lipopolysaccharide-associated toxicity, in C57BL/6 mice using both acute and chronic lung infection models. Mice received two or three doses at various intervals, and safety was assessed through clinical and weight monitoring. A high-dose, 2-administration intranasal regimen protected against acute infection but induced transient weight loss, while a lower-dose, 3-administration regimen minimized toxicity. Immunized mice exhibited robust IL-17A production and increased IFN-γ responses. Following challenge with mucoid strains, vaccinated mice showed reduced lung bacterial loads, suggesting a partial protection against chronic infection. These findings support the potential of auxotrophic live vaccines to mitigate disease severity in patients with cystic fibrosis.

## 1 Introduction

*Pseudomonas aeruginosa* is a common opportunistic pathogen that affects immunocompromised individuals as well as healthy individuals whose skin or mucosal barriers are disrupted by wounds, burns, catheterization, or endotracheal intubation. The bacterium is capable of causing a broad spectrum of infections, ranging from mild conditions such as folliculitis to severe systemic diseases, including pneumonia and bacteremia (1). Globally, lower respiratory tract infections were the third leading cause of death in 2019, accounting for approximately 4 million fatalities, including an estimated 233,000 deaths attributed to *P. aeruginosa* (2). These infections manifest either as acute, affecting individuals with pre-existing immunodeficiency or mechanically ventilated patients, or as chronic, involving recurrent episodes in patients with cystic fibrosis (CF) or chronic obstructive pulmonary disease (COPD) (3). Previous studies reported that the prevalence of *P. aeruginosa* in adults with CF varies between 31% and 47% (4), significantly contributing to increased morbidity and mortality in these patients.

During acute infection, *P. aeruginosa* can elicit a robust inflammatory response. However, in patients with structural lung damage, such as those with CF or COPD, it can persist and establish chronic infections (5). This chronicity is often associated with its ability to form biofilms, which are clusters of microorganisms irreversibly attached to biotic or abiotic surfaces and embedded in an extracellular polymeric matrix (6). This biofilm-mediated growth enables *P. aeruginosa* to evade host immune defenses and resist the action of antimicrobial agents. Chronic infection is characterized by recurrent or intermittent colonization, typically following the establishment of bacterial reservoirs such as in the paranasal sinuses (7). Nutrient and space limitation, hypoxia due to mucus accumulation, antibiotic exposure, and the infiltration of neutrophils and other immune cells trigger bacterial stress responses. Ultimately, these selective pressures drive mutations in *P. aeruginosa* that promote genetic and phenotypic adaptation (8).

During bacterial adaptation, *P. aeruginosa* undergoes diverse modifications, including the emergence of small-colony and rough small-colony variants, auxotrophy, alginate hyperproduction, and loss of flagella and pili to support a sessile biofilm-associated lifestyle (9, 10). Additional changes include alterations in lipopolysaccharide (LPS) composition, reduced cytotoxicity due to the loss of the type III secretion system, attenuated quorum-sensing communication, and a metabolic shift in iron acquisition from siderophores to hemoglobin (8, 9, 11, 12). Together, these adaptations facilitate the establishment of chronic infections, contributing to a progressive lung function decline and, consequently, increased morbidity (10).

*P. aeruginosa* belongs to the ESKAPE group (*Enterococcus faecium*, *Staphylococcus aureus*, *Klebsiella pneumoniae*, *Acinetobacter baumannii*, *P. aeruginosa* and *Enterobacter* spp.), which is responsible for the majority of hospital-acquired infections and exhibits remarkable resistance to antimicrobial agents (13, 14). In response to the growing threat of Antimicrobial Resistance (AMR), the World Health Organization (WHO) has included ESKAPE bacteria in its list of 15 priority pathogens that urgently require the development of new therapies (15, 16). Among these, carbapenem-resistant *P. aeruginosa* is classified as a “high priority” pathogen (Priority 2). Given the rising prevalence of AMR, developing alternative therapeutic approaches with novel mechanisms of action is critical. However, recent research has largely focused on modifying existing antibiotics rather than developing entirely new drug classes (17). In this context, vaccines have emerged as a promising tool against multidrug-resistant bacteria. Vaccination has proven effective in reducing antibiotic use for bacterial and viral infections, thereby mitigating the selective pressure that drives the emergence of antibiotic-resistant strains (18–20).

Among the various immunization strategies investigated for *P. aeruginosa*, live attenuated vaccines, particularly auxotrophic mutants, constitute an attractive approach due to their strong immunogenicity and ability to induce robust cellular immune responses, which offer a key advantage over inactivated or subunit-based vaccines (21–23). Auxotrophic vaccines based on the inactivation of genes essential for cell wall synthesis exhibit self-limiting growth *in vivo*, minimizing the risk of disease, transmission, or reversion to virulence (22, 24). Moreover, issues related to LPS-associated reactogenicity have been partially addressed by inactivating enzymes involved in LPS lipid modification, further enhancing vaccine safety (25).

To evaluate vaccine-induced protection against *P. aeruginosa*, we utilized a previously described murine model of chronic infection (26). In this model, C57BL/6 mice are intratracheally (IT) inoculated with *P. aeruginosa* embedded in agar particles. Immobilizing agents such as agar enable bacterial growth under microaerobic or anaerobic conditions that mimic the mucus-rich airways of individuals with CF. This environment promotes slow, persistent bacterial proliferation within microcolonies, driving the development of chronic infection and subsequent pulmonary inflammation (27). The incidence of chronic infection in this model depends on several factors, including the mouse strain, inoculum size, and the specific bacterial strain used (26). Notably, C57BL/6 mice exhibit higher rates of chronic infection alongside lower mortality and slower bacterial clearance than other mouse strains, making them the preferred model for chronic lung infection studies (27).

This study aims to evaluate the safety and protective efficacy of these auxotrophic vaccine candidates, along with a derivative strain exhibiting reduced reactogenicity, in C57BL/6 mouse models of acute and chronic respiratory infection.

## 2 Materials and methods

### 2.1 Bacterial strains and growth conditions

The bacterial strains used in this study are listed in **Table 1**. Bacteria were routinely cultured in Luria-Bertani (LB) broth (10 g/L tryptone, 5 g/L yeast extract and 10 g/L NaCl) with shaking (180 rpm) at 37 °C or on LB plates containing 2% agar. *P. aeruginosa* PAO1 Δ*murI* was cultivated in LB medium supplemented with 10 mM D-glutamate (Sigma-Aldrich, USA). *P. aeruginosa* PAO1 Δ*murI* Δ*alr* Δ*dadX* (referred to as PAO1 ΔΔΔ) and its derivative mutant PAO1 ΔΔΔ Δ*htrB1* Δ*pagP* were cultured in LB medium supplemented with 8 mM D-glutamate and 6 mM D-alanine (Sigma-Aldrich, USA). Mucoid *P. aeruginosa* strains, isolated from individuals with CF, were used in the chronic infection model. These strains were grown in tryptic soy broth (TSB) at 37 °C with shaking (180 rpm) or on tryptic soy agar supplemented with 5% sheep blood (TSA-S, Thermo Fisher Scientific, USA). Ampicillin (Ap; Sigma-Aldrich, USA) was added at 50 µg/mL when required.

**Table 1:** Bacterial strains used in this study.

Strain	Relevant features	Reference
*P. aeruginosa*		
PAO1	Reference strain. Isolate from a patient with a burn wound infection.	CECT 4122 (65)
PAO1 Δ*murI*	PAO1 derivative lacking *murI* (Locus tag PA4662). Auxotroph for D-glutamate.	(22)
PAO1 Δ*murI* Δ*alr* Δ*dadX* (= PAO1 ΔΔΔ)	PAO1 Δ*murI* derivative lacking *alr* and *dadX* genes (Locus tag PA4930 and PA5302, respectively). Auxotroph for D-glutamate and D-alanine.	(24)
PAO1 ΔΔΔ Δ*htrB1* Δ*pagP*	PAO1 ΔΔΔ derivative lacking *htrB1* and *pagP* genes (Locus tag PA0011 and PA1343, respectively).	(25)
CF-51419182 (strain A)	Isolate from the sputum of an individual with CF and bronchial infection at CHUAC, Spain; mucoid phenotype.	Laboratory collection
CF-51429475 (strain E)	Isolate from the sputum of an individual with CF and bronchial infection at CHUAC, Spain; mucoid phenotype.	Laboratory collection
DK1-NH57388A	Isolate from sputum of an individual with CF in Denmark (CF86); mucoid phenotype.	(35) Kindly provided by Dr. Niels Høiby (University of Copenhagen, Denmark)
LES400	Liverpool epidemic strain from an individual with CF and chronic infection.	(34)
PA14	Hypervirulent strain isolated from an individual with a burn wound infection.	(66)

### 2.2 Heat-killed bacterial cells

For heat inactivation, bacteria were grown in LB or TSB to an optical density at 600 nm (OD_600_) of 0.7. Then, bacteria were harvested by centrifugation, and the pellets were resuspended in sterile saline solution (0.9% NaCl). Aliquots of 1 mL were incubated at 100 °C for 2 h. Bacterial viability was assessed before and after heat treatment by plating onto LB agar and incubating at 37 °C for 16 h to confirm complete inactivation.

### 2.3 Animal experiments

Animal experiments were conducted in strict compliance with European Union legislation (Directive 2010/63/EU) and relevant national regulations (RD 53/2013) for the protection of animals used for scientific purposes. All the procedures were reviewed and approved by the Animal Experimentation Ethics Committee of CHUAC (Project Reference Numbers 15002/2017/01 and 15002/2021/005). C57BL/6 mice (6–14 weeks old) were either purchased from Envigo RMS Holding Corp (now Inotiv Inc.) or provided by the Training Technology Center of the *Xerencia de Xestión Integrada A Coruña* (CTF-XXIAC). Animals sourced from Envigo RMS were acclimatized for 10 days prior experimentation. All mice were housed in specific pathogen-free facilities at the CTF-XXIAC, under standardized light-controlled conditions (12-h light/dark cycle), with unrestricted access to pelleted rodent chow and water. Male and female mice were randomly assigned to experimental or control groups within each sex, with 4–6 animals housed per cage.

### 2.4 Inoculum preparation for immunization and infection

To prepare the bacterial inocula, cultures were incubated in the appropriate medium at 37 °C with shaking for 16 h. Overnight cultures were diluted 1:50 in fresh medium and grown to an OD_600_ of 0.7. Bacteria were harvested by centrifugation (5,000 × g, 15 min, 4 °C), washed three times with sterile saline solution, and resuspended to the target concentration for *in vivo* use. Serial dilutions of the suspensions were plated onto the appropriate culture media to determine the number of colony-forming units (CFU) administered per mouse.

For immunogenicity assessments, intradermal (ID) and intranasal (IN) administration routes were employed. Mice were anesthetized with sevoflurane prior to each procedure. Immunization schedules were optimized based on previous protocols (25, 28). For the acute infection model, two IN doses were administered 14 days apart; for the chronic model, three doses were given at 7-day intervals via either ID or IN route.

### 2.5 Preparation of *P. aeruginosa* embedded in agar beads

Agar beads were prepared following the method described by Facchini et al. (2014) (26) with minor modifications. Briefly, mucoid *P. aeruginosa* were cultured in TSB at 37 °C with agitation (180 rpm) for 16 h. Bacterial cultures were then diluted 1:10 in fresh medium and grown to an OD_600_ of 0.7. Cells were harvested by centrifugation (5,000 × *g*, 20 min, 4 °C), washed with sterile phosphate-buffered saline (PBS), and resuspended in 1 mL PBS. This suspension was mixed with 9 mL TSA maintained at 50 °C and added dropwise into 150 mL of mineral oil (Sigma-Aldrich, USA) at 50 °C under continuous stirring at 700 rpm. The mixture was then transferred to an ice bath and gently agitated at 30 rpm for 35 min to allow bead formation (50-100 µm diameter). The bead suspension was centrifuged (2,700 rpm × *g*, 15 min, 4 °C), washed five times with sterile PBS, resuspended in a final volume of 25 mL PBS, and stored at 4 °C until use. The bacterial concentration within the beads (CFU/mL) was determined by plating on TSA and incubating at 37 °C for 24 h, and then adjusted with PBS to a target dose of ~1 × 10^6^ CFU per 50 µL.

### 2.6 Acute lung infection model

To induce acute lung infection, C57BL/6 mice were anesthetized with inhaled sevoflurane (Baxter International, Inc.) and IN inoculated with 20 µL of a bacterial suspension containing 1.5 × 10^6^ CFU of the hypervirulent *P. aeruginosa* PA14 strain, evenly distributed between both nostrils. To minimize expulsion of the inoculum and to facilitate the establishment of acute pneumonia, mice were maintained under anesthesia for an additional 2 min after inoculation.

Mice were monitored daily with individualized follow-ups. Clinical signs (*e.g.*, lethargy, piloerection, nasal discharge, and skin lesions) were recorded, and body weight was tracked as percentage change from baseline (day 0). Humane endpoints were applied in cases of > 25% weight loss or severe clinical distress, in accordance with established guidelines (29, 30). Euthanasia was performed by intraperitoneal (IP) administration of a thiopental anesthetic overdose (300 mg/kg; B Braun, Spain). Death was confirmed by the complete absence of a withdrawal reflex following a deep toe pinch, after which cervical dislocation was performed.

### 2.7 Chronic lung infection model

To establish chronic lung infection, C57BL/6 mice were inoculated IT with mucoid *P. aeruginosa* strains embedded in agar beads, following a previously described protocol (26). During IT administration, mice were placed on an inclined platform and anesthetized under a continuous flow of sevoflurane. Guided by an endoscope, 50 µL of the *P. aeruginosa*-agar bead suspension (5 × 10^5^ – 2 × 10^6^ CFU) was delivered using a Hamilton^®^ syringe fitted with an olive-tip cannula (25 G, 7/8’’). Immediately after inoculation, mice were administered an IP anesthetic cocktail consisting of 10 µL of medetomidine hydrochloride (1 mg/L; Ecuphar, Spain) and 15 µL of ketamine (50 mg/mL; Pfizer Spain), and maintained in a vertical position for 20 min to prevent expulsion of the inoculum. Anesthesia was subsequently reversed by IP injection of 10 µL of atipamezole hydrochloride (5 mg/mL; Ecuphar, Spain).

### 2.8 Preparation of lung homogenates for bacterial counts

Mice were euthanized by IP administration of a thiopental overdose (300 mg/kg). Death was confirmed by the absence of a withdrawal response to a deep toe pinch, together with cessation of respiration and heartbeat and the onset of rigor mortis. The lungs were then aseptically removed, weighted, and homogenized in sterile saline solution using a Mixer Mill MM200 (Restch) at 25 Hz for 20 min. For bacterial counts, lung homogenates were serially diluted and plated on TSA or LB agar containing ampicillin and incubated at 37 °C for 24 h. The results were expressed as CFU per gram of lung tissue (CFU/g lung).

### 2.9 Preparation of single-cell suspensions from mouse lung tissue

Lungs were aseptically excised and cut into small pieces (1–3 mm), which were incubated in RPMI-1640 medium containing 0.5% collagenase D (Roche Diagnostics, GmbH, Germany) at 37 °C for 30 min. The digested tissue was mechanically dissociated using a syringe plunger and filtered through a 100 µm cell strainer (Corning, USA). The resulting cell suspension was diluted with 3 mL of PBE buffer (PBS, 2 mM EDTA, 0.5% BSA) and centrifuged at 70 × *g* and 4 °C for 1 min. The supernatant was transferred to a new tube and cells were harvested by centrifugation (300 × *g*, 10 min, 4 °C). The cell pellet was resuspended in 1 mL of ACK lysis buffer (150 mM NH_4_Cl, 10 mM KHCO_3_, 0.1 mM EDTA, pH 7.2) and incubated at room temperature for 4 min to lyse erythrocytes. After adding 5 mL of RPMI-1640, the mixture was centrifuged again (300 × *g*, 10 min, 4 °C) and resuspended in 1 mL of complete RPMI-1640 medium [supplemented with 10 mM HEPES, 200 IU/mL penicillin, 200 μg/mL streptomycin, 50 μM 2-mercaptoethanol, and 10% fetal bovine serum (FBS)]. Cells counts were determined from a 1:10 dilution using a CytoSMART Cell Counter, and the suspension was adjusted to a final concentration of 1 × 10^7^ cells/mL.

### 2.10 Preparation of single-cell suspensions from mouse spleen

Spleens were mechanically dissociated by gently pressing the tissue through a 100 μm cell strainer using the rubber plunger of a sterile syringe. The resulting cell suspension was adjusted to a final volume of 4 mL with PBS. A 2 mL aliquot was centrifuged (300 × *g*, 10 min, 4 °C), and the cell pellet was resuspended in 1 mL of ACK lysis buffer and incubated at room temperature for 4 min. After the addition of 5 mL RPMI-1640, cells were harvested by centrifugation (300 × *g*, 10 min, 4 °C) and resuspended in 2 mL of complete RPMI-1640 medium. Cell counts were determined from a 1:10 dilution in complete medium using a CytoSMART Cell Counter, and the final cell concentration was adjusted to 1 × 10^7^ cells/mL.

### 2.11 *Ex vivo* antigenic stimulation and cytokine quantification

Single-cell suspensions from lungs or spleens, prepared in complete RPMI-1640, were seeded into round-bottom 96-well plates at a density of 5 × 10^5^ cells per well. Cells were stimulated at a 1:1 ratio with heat-inactivated *P. aeruginosa* PAO1 or DK1-NH57388A strains (5 × 10^5^ CFU) and incubated for 3 days at 37 °C, 5% CO_2_. Negative control wells received RPMI-1640 medium alone. All experiments were performed in duplicate. After incubation, plates were centrifuged (300 × *g*, 10 min), and the supernatants were transferred to 96-well V-bottom plates and stored at –80 °C until analysis. Soluble cytokines in the culture supernatants were quantified using commercial ELISA kits according to the manufacturer’s instructions.

### 2.12 Flow cytometric analysis

Cytokine-producing CD4^+^ T cells from splenic and pulmonary single-cell suspensions were identified by flow cytometry, as previously described (28). Cells (1 × 10^6^) were seeded into 96-well plates (Costar, Corning, USA) and stimulated with either 50 ng/mL phorbol 12-myristate 13-acetate (PMA) and 500 ng/mL ionomycin in the presence of 10 μg/mL Brefeldin A (all from Sigma-Aldrich, USA), or heat inactivated *P. aeruginosa* PAO1 (5 × 10^5^ CFU), and incubated at 37 °C with 5% CO_2_ for 4–5 hours. Following incubation, cells were harvested by centrifugation (670 × *g*, 2 min). For extracellular staining, cells were incubated with the viability dye FVD–APC-eF780 (Allophycocyanin-eFluor™ conjugate, eBioscience, USA) diluted 1:1000 in PBS for 20 min at 4 °C to exclude dead cells. Cells were washed with PBS (670 × *g*, 2 min) and incubated with anti-mouse CD16/CD32 (Clone 2.4G2; Rat IgG2b; κ isotype; BD Biosciences, USA) to block FcγR receptors, together with anti-mouse CD4 BV421 (Clone RM4-5; Rat IgG2a, κ isotype; BD Biosciences, USA) (1:200), and anti-mouse CD3 eF506 (Clone 17A2; Rat IgG2b; κ isotype; eBioscience, USA) (1:200), diluted in FACS buffer (2 mM EDTA, 4% heat-inactivated FBS in PBS), for 25 min at 4 °C.

For intracellular staining, following extracellular staining, cells were fixed with 2% formaldehyde in PBS for 25 min at room temperature, and permeabilized with Permeabilization Buffer [PB; FACS buffer containing 0.05% saponin (Sigma-Aldrich, USA)] for 10 min. Fcγ receptors were blocked with anti-mouse CD16/CD32 (Clone 2.4G2; Rat IgG2b; κ isotype; BD Biosciences, USA) for 15 min at room temperature, followed by intracellular staining using the following antibodies diluted in PB: anti-mouse IL-17A FITC (Clone TC11-18H10.1; Rat IgG1; κ isotype; BioLegend, USA) (1:200), anti-mouse IL-4 PE-Cy7 (Clone 11D11; Rat IgG1; κ isotype; BD Biosciences, USA) (1:200), anti-mouse IFN-γ APC (Clone XMG1.2; Rat IgG1; κ isotype; BioLegend, USA) (1:200), and anti-mouse IL-10 PE (Clone JES5-16E3; Rat IgG2b; κ isotype; BioLegend, USA) (1:100), incubated for 30 min at room temperature. Finally, samples were washed twice with PB (670 × *g*, 2 min) and resuspended in FACS buffer at 4 °C until acquisition.

Non-stimulated lung myeloid cells were analyzed by flow cytometry following surface antigen staining. Cells were incubated with the viability dye FVD–APC-eF780 and blocked with purified anti-mouse CD16/CD32 prior to incubation with anti-mouse F4/80 Phycoerythrin (PE)-conjugate (clone BM8), anti-mouse I-A/I-E Peridinin Chlorophyll Protein Complex (PerCP/Cy5.5)-conjugate (clone M5/114.15.2), anti-mouse CD45 APC-conjugate (clone 30-F11), anti-mouse CD11c BV421-conjugate (clone N418), anti-mouse Ly6G FITC-conjugate (clone 1A8) and anti-mouse Ly6C PE-Cy7-conjugate (clone HK1.4) (all from BioLegend); anti-mouse CD11b BV510-conjugate (clone M1/70) (from BD Biosciences).

Flow cytometry was performed using a BD FACSCanto™ II cytometer (BD Biosciences, USA) with BD FACSDiva™ software, acquiring at least 150,000 live events per sample. Fluorescence minus one (FMO) controls were used to define gating boundaries, and isotype controls were included to assess non-specific staining.

### 2.13 ELISA

The levels of IFN-γ, IL-4, IL-10, and IL-17A in cell culture supernatants were assessed using the following commercial kits: BioLegend MAX Standard Set (for IL-4), R&D Systems DuoSet^®^ ELISA (for IL-10), and Invitrogen ELISA Ready-Set-Go (for IFN-γ and IL-17A), according to the manufacturers’ instructions.

### 2.14 Histopathological analysis

Pulmonary tissues were fixed in 10% buffered formalin and embedded in paraffin. Serial consecutive sections (2 µm thick) were cut and routinely stained with hematoxylin and eosin (H&E), according to standard protocols. Slides were examined using a Nikon Eclipse E600 microscope equipped with ×2, ×4, ×10 and ×40 objectives, and images were captured with a Nikon Digital Sight DS-5M digital camera equipped with a Nikon PLAN UW 2X/0.06 lens. Histopathological evaluation was performed by a pathologist blinded to the experimental groups.

Histopathological alterations were assessed using a semi-quantitative severity scoring system. Lesions were graded according to both their extent and distribution as follows: score 0 (absent), no detectable alterations; score 1 (minimal), slight, focal changes involving up to 5% of the examined tissue; score 2 (mild), focal to multifocal alterations affecting 6–25% of the tissue; score 3 (moderate), multifocal to diffuse changes involving approximately 26–50% of the tissue; score 4 (marked), extensive alterations affecting 51–80% of the tissue; and score 5 (severe), marked, widespread alterations representing a dominant feature and involving 81–100% of the examined tissue.

Additionally, the type of inflammatory infiltrate was recorded, along with other histopathological features, including oedema, alveolar consolidation, necrosis, bronchial and bronchiolar epithelial hyperplasia, and pleural changes (including pleural reactivity and pleuritis).

### 2.15 Statistical analysis and software

GraphPad Prism (version 8.4.2) was used to generate most of the graphs and perform statistical analysis. Statistical analyses included the Mann-Whitney U test for comparisons between two independent groups and the Kruskal-Wallis test with Dunn’s *post hoc* multiple-comparisons test for comparisons among three or more groups. When deviations from normality were minor, data were analyzed using one-way ANOVA followed by Tukey’s multiple-comparisons test. Survival analyses were performed using the log-rank Mantel-Cox test, mean comparisons used the unpaired *t*-test, and significance levels were adjusted with the Holm-Sidak method. For experiments with individual longitudinal tracking, weight changes were analyzed using a two-way repeated-measures ANOVA with Dunnett’s multiple comparisons test or a linear mixed-effects model, followed by Tukey’s *post hoc* multiple-comparisons test. Data obtained from flow cytometry analyses were processed using FlowJo_V10.9.0 software (TreeStar).

## 3 Results

### 3.1 Intranasal immunization with PAO1 Δ*murI* protects C57BL/6 mice against acute pneumonia

**Figure 1:**
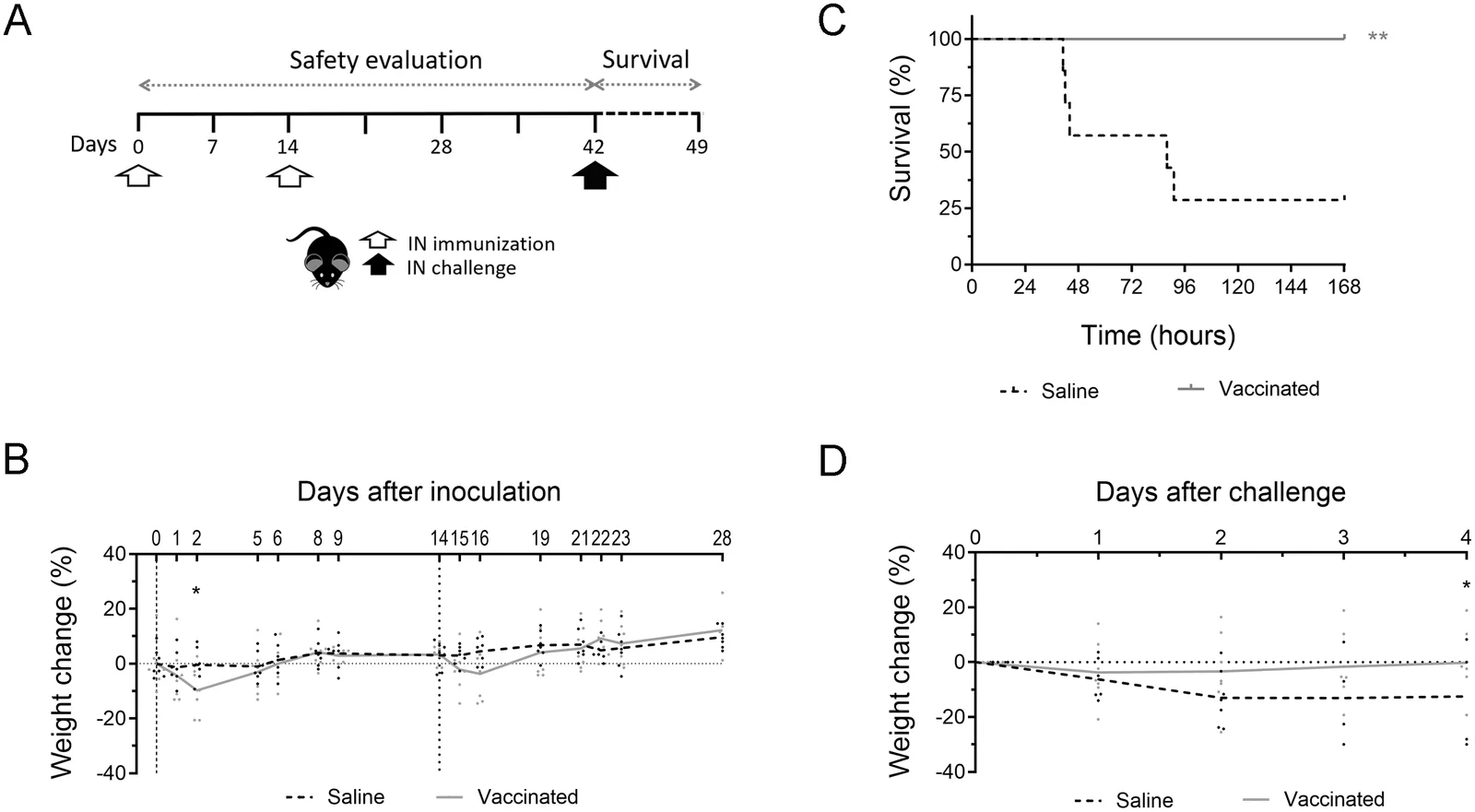
Safety and efficacy of PAO1 Δ*murI* using an acute infection model. **(A)** Two-dose immunization and challenge schedule. **(B)** Body weight change (%) in female C57BL/6 mice (*n* = 7) following IN immunization with PAO1 Δ*murI* (2.5 × 10^8^ CFU) or saline administration. Individual mice are shown, with group means connected by lines. *p < 0.05, compared with the saline control group (multiple *t* test corrected using the Holm-Sidak method). **(C)** Mice survival after IN immunization with PAO1 Δ*murI* (as in B) and IN challenge with PA14 strain (1.5 × 10^6^ CFU), four weeks after immunization, according to schedule **(A)** **p < 0.01, Log rank (Mantel-Cox) test. **(D)** Body weight change (%) after IN challenge in previously immunized mice or saline administered. Individual mice are shown, with group means connected by lines. *p < 0.05, compared with the saline control group (mixed-effects model with Tukey’s multiple-comparisons test).

The immunogenicity and protective efficacy of the D-glutamate auxotrophic vaccine strain, PAO1 Δ*murI*, have been extensively evaluated in sepsis (22) and acute pneumonia (28) models using BALB/c mice, which are characterized by a Th2-prone immune response (31–33). To account for potential strain-specific immune bias, protection was also demonstrated in Th1-skewed C57BL/6 mice (31–33) using acute systemic infection models following IP and intramuscular vaccination (22). In the present study, we assessed the safety and efficacy of PAO1 Δ*murI* in an acute pneumonia model using C57BL/6 mice. Safety was evaluated by monitoring body weight following IN vaccination (**Figure 1A**). Mice (*n* = 7 per group) received two IN administrations of approximately 2.5 × 10^8^ CFU, given 14 days apart. After each administration, mice experienced transient weight loss (up to 10%), which was fully recovered within 7–8 days (**Figure 1B**). Protective efficacy was then evaluated in a murine model of acute lung infection. Four weeks after the final immunization (day 42), mice were IN-challenged with the hypervirulent PA14 strain (1.5 × 10^6^ CFU), and survival was monitored for 7 days (**Figure 1A**). All vaccinated mice survived, whereas unvaccinated controls exhibited approximately 15% weight loss and five out of seven (71%) ultimately succumbed to the infection (**Figures 1C, D**). These findings demonstrate that PAO1 Δ*murI*, despite causing transient weight loss, confers protection against hypervirulent PA14 challenge in C57BL/6 mice, consistent with previous observations in the BALB/c background (28).

### 3.2 Optimization of the chronic lung infection model in C57BL/6 mice

Initially, *P. aeruginosa* strains with a mucoid morphotype were obtained from individuals with CF and chronic infections. To verify the stability of the mucoid phenotype, several passages were performed on TSA-S plates, followed by subcultures in liquid LB medium. From this process, the following *P. aeruginosa* strains were selected: strain A (isolate no. 51419182) and strain E (isolate no. 51429475), both from CHUAC; the epidemic strain LES400 from Liverpool (34); and strain DK1-NH57388A (35) (**Table 1**; **Supplementary Figure 1A**).

To optimize the chronic lung infection model, the selected strains were embedded in agar particles (**Supplementary Figure 1B**) and used to infect C57BL/6 mice (*n* = 14−21 per group) via the IT route, as described in Materials and Methods. Other infection-related variables were assessed for their association with acute mortality and pulmonary colonization (**Supplementary Table 1**). At 11 days post-infection, more than 70% of mice showed pulmonary colonization across all tested bacterial strains, with the exception of strain E that displayed reduced colonization rates. Correlation analysis revealed a weak negative association between initial body weight and acute mortality after challenge (Pearson’s r = −0.2572), and a weak positive association between body weight and chronic colonization (r = 0.2957). No significant differences were observed with respect to sex. In contrast, inoculum dose showed moderate positive correlations with both acute mortality (r = 0.5506) and chronic colonization (r = 0.5031), suggesting a potential dose-dependent effect on disease severity and persistence. Although these correlation coefficients suggest weak-to-moderate associations, none of the analyses reached statistical significance, likely due to the limited sample size and reduced statistical power.

### 3.3 Evaluation of the D-glutamate auxotrophic vaccine strain (PAO1 Δ*murI*) in a chronic lung infection model

**Figure 2:**
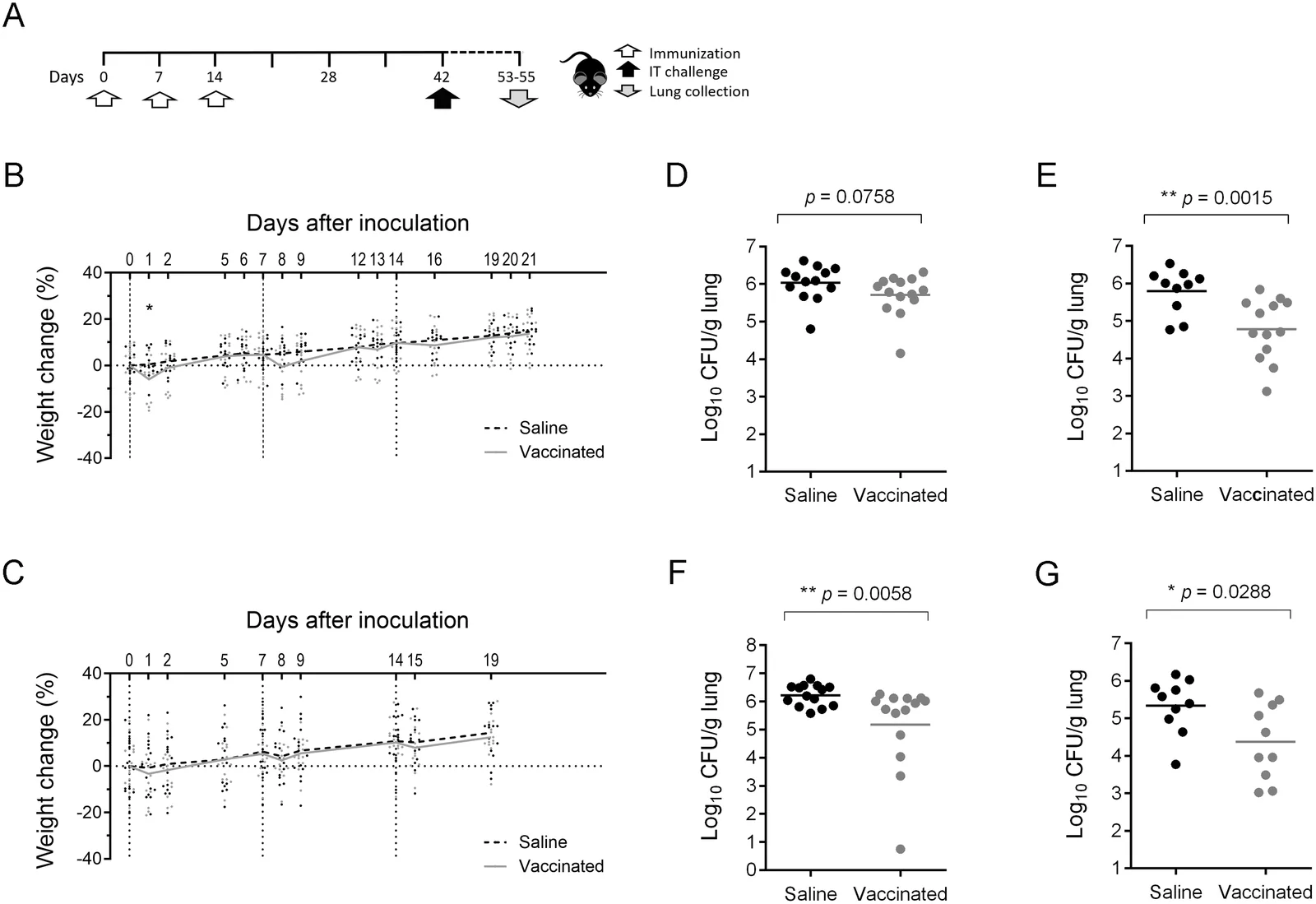
Safety and protective efficacy using the chronic lung infection model. **(A)** Immunization schedule, challenge, and lung collection. **(B, C)** Safety assessment following ID and IN immunization with PAO1 Δ*murI* using a three-dose regimen. Percent change in body weight of male C57BL/6 mice (*n* = 13−16) after **(B)** ID (8 × 10^7^ CFU) or **(C)** IN (3 × 10^7^ CFU) immunization. Individual mice are shown, with group means connected by lines. *p < 0.05 (multiple *t* tests corrected using the Holm-Sidak method). **(D-G)** Protective efficacy of PAO1 Δ*murI* immunization against chronic lung infection in mice. C57BL/6 mice were immunized with PAO1 Δ*murI* via either ID (8 × 10^7^ CFU; *n* = 10−14) **(D, E)** or IN (1.5 × 10^7^ CFU; *n* = 10−14) **(F, G)** route. Four weeks after immunization, mice were challenged IT with strain A **(D, F)** or DK1-NH57388A **(E, G)** embedded in agar beads (~1 × 10^6^ CFU). Lung bacterial loads were determined 11−13 days post-challenge in vaccinated and control mice. Horizontal lines indicate mean values. *p < 0.05, **p < 0.01, Mann-Whitney U test, compared with the saline control group.

To evaluate the safety of PAO1 Δ*murI*, male C57BL/6 mice were immunized using a three-dose schedule at 7-day intervals via two different administration routes (**Figure 2A**). One group received three ID doses (8 × 10^7^ CFU, *n* = 13), while the second group received three IN doses (3 × 10^7^ CFU, *n* = 15). The IN dose was approximately one-eighth of that used in the acute infection model to minimize route-associated toxicity (**Figure 1**). Safety was evaluated by monitoring body weight throughout the study and examining local reactions at the inoculation sites. Although a transient reduction in body weight occurred within 3 days post-vaccination, this decrease remained below 5% relative to baseline and showed no significant difference compared with the saline-treated control group (**Figures 2B, C**). Following ID administration, small transient skin ulcers developed at the injection site; however, these lesions resolved spontaneously within one week.

To assess the protective efficacy of PAO1 Δ*murI* in a chronic lung infection model, C57BL/6 mice were immunized as described above: ID (2 groups, *n* = 10–14 mice, 8 × 10^7^ CFU) and IN (2 groups, *n* = 10–14 mice, 1.5 × 10^7^ CFU). Four weeks after the final immunization (day 42), mice were challenged IT with either strain A or strain DK1-NH57388A embedded in agar beads. After an 11–13 day monitoring period, lung bacterial loads were quantified (**Figure 2A**). Following challenge with strain A, ID immunization failed to significantly reduce bacterial burden compared to non-immunized controls (**Figure 2D**). In contrast, IN-immunized mice showed significant reduction in pulmonary bacterial burden of approximately 1-log (*p* < 0.01, Mann-Whitney U test) (**Figure 2F**). In the chronic infection model using strain DK1-NH57388A, both ID and IN vaccination conferred significant protection, achieving approximately 1-log reductions in bacterial loads compared with controls (*p* < 0.05 and *p* < 0.01, respectively; Mann-Whitney U test) (**Figures 2E, G**).

### 3.4 Comparative analysis of the double auxotrophic vaccine strain (PAO1 ΔΔΔ) and its LPS-modified derivative, PAO1 ΔΔΔ Δ*hrtB1* Δ*pagP*

We compared the double auxotrophic vaccine prototype PAO1 ΔΔΔ with its LPS-modified derivative PAO1 ΔΔΔ Δ*htrB1* Δ*pagP*, which is deficient in the acyltransferase HtrB1 and the palmitoyltransferase PagP. This derivative showed enhanced attenuation of virulence in murine models and reduced cellular endotoxicity (25), supporting its potential as a vaccine candidate against *P. aeruginosa* infections.

To assess safety, protective efficacy, and immunogenicity, C57BL/6 mice (*n* = 28 per group) were immunized with either PAO1 ΔΔΔ or PAO1 ΔΔΔ Δ*htrB1* Δ*pagP* via ID or IN routes and compared to saline controls. The immunization regimen consisted of three doses: 1 × 10^8^ CFU for ID and 2.5 × 10^7^ CFU for IN. Four weeks post-immunization, lungs and spleens from a subset of mice (*n* = 8 per group) were analyzed for immune response, while the remaining animals (*n* = 20) were challenged IT with *P. aeruginosa* DK1-NH57388A (1 × 10^6^ CFU) embedded in agar beads. Thirteen days after challenge, pulmonary bacterial loads (*n* = 12) and immune responses in the lungs and spleens (*n* = 8) were evaluated.

#### 3.4.1 *In vivo* safety assessment of the vaccine strains after ID and IN administration in C57BL/6 mice

**Figure 3:**
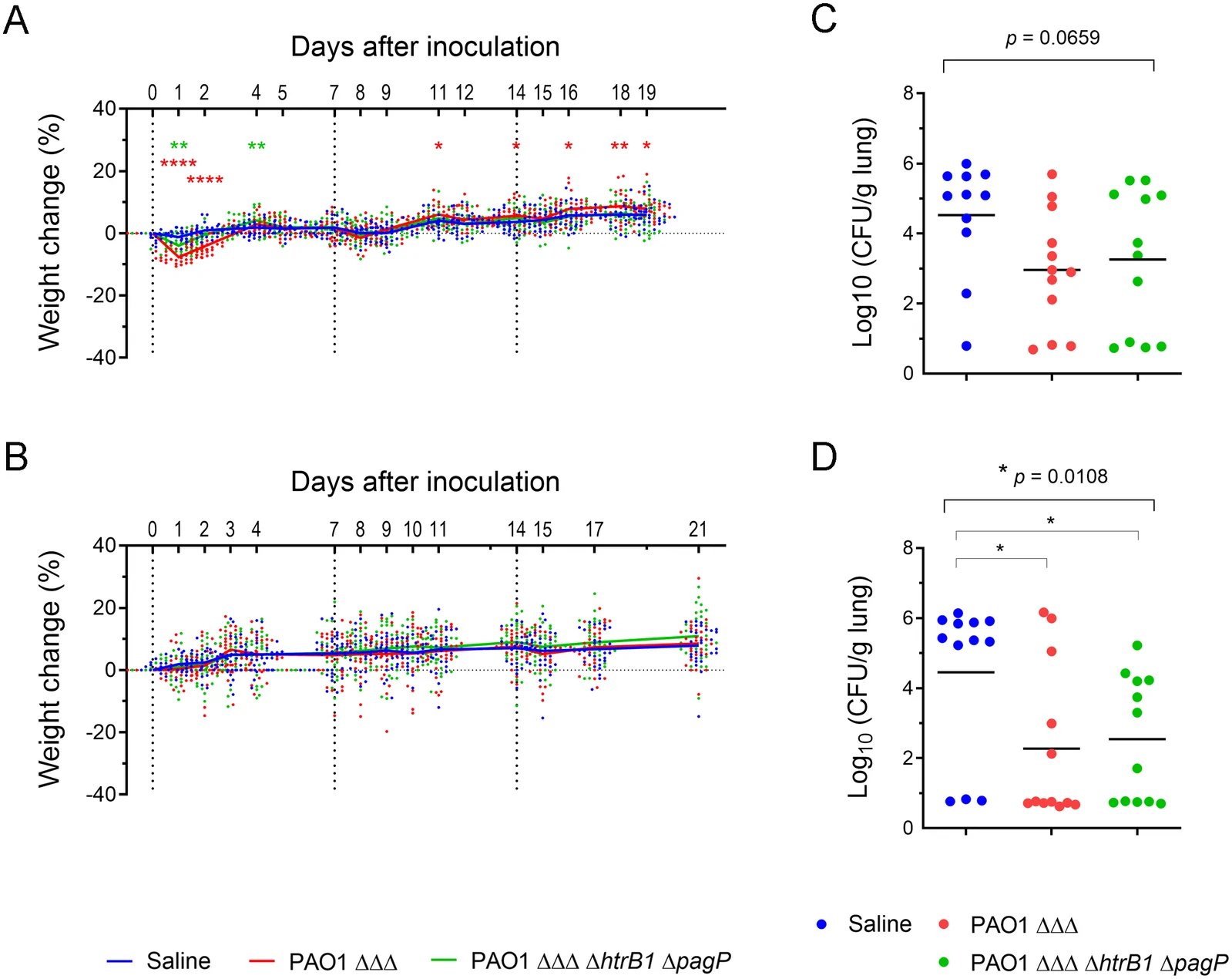
Safety assessment and protective efficacy against chronic lung infection following immunization with PAO1 ΔΔΔ and PAO1 ΔΔΔ Δ*htrB1* Δ*pagP*. **(A, B)** Body weight change (%) in C57BL/6 mice (*n* = 28) after **(A)** ID (1 × 10^8^ CFU) or **(B)** IN (2.5 × 10^7^ CFU) immunization using a three-dose schedule. Dashed lines indicate immunization days (0, 7 and 14). Individual mice are shown, with group means connected by lines. *p < 0.05, **p < 0.01, ****p < 0.0001, compared with the saline control group (two-way repeated-measures ANOVA with Dunnett’s multiple comparisons test. **(C, D)** Four weeks after the last ID **(C)** or IN **(D)** vaccine dose, mice (*n* = 12 per group) were IT challenged with DK1-NH57388A embedded in agar beads (1 × 10^6^ CFU). Thirteen days post-challenge, lung bacterial loads were determined. Overall group differences were assessed using Kruskal-Wallis test, followed by Dunn’s *post hoc* multiple-comparisons test (*p < 0.05).

Following administration of the vaccine candidates via ID and IN routes, body weight and clinical parameters were monitored periodically, including physical appearance (*e.g.*, piloerection, grooming), behavioral changes (activity levels, response to stimuli), and respiratory patterns. In the ID-vaccinated groups, a transient weight loss was observed the day after inoculation with both PAO1 ΔΔΔ and PAO1 ΔΔΔ Δ*htrB1* Δ*pagP*. This reduction accounted for 8% and 4% of the initial mean body weight, respectively, and resolved spontaneously thereafter (**Figure 3A**). Mice remained in good overall condition throughout the study, although local lesions were developed at the injection site. Measurement of the wound area demonstrated that lesions induced by the modified LPS mutant were smaller than those induced by PAO1 ΔΔΔ (**Supplementary Figure 2A**). Histopathological examination of the skin after healing revealed no relevant alterations, and no significant differences were observed among the experimental groups. The overall skin architecture was preserved across all groups, displaying normal stratification of the epidermis and dermis, alongside the presence of hair follicles at various stages of development (**Supplementary Figure 3A**).

In contrast, administration of either strain via the IN route at a dose of 2.5 × 10^7^ CFU resulted in minimal weight loss and no apparent clinical signs (**Figure 3B**). Increasing the inoculum to 8 × 10^8^ CFU revealed clear differences between the two strains; the LPS-modified derivative PAO1 ΔΔΔ Δ*htrB1* Δ*pagP* exhibited reduced reactogenicity compared with the double auxotrophic vaccine prototype PAO1 ΔΔΔ (**Supplementary Figure 2B**).

Mild hyperplasia of bronchus-associated lymphoid tissue (BALT) was observed in mice, being more prominent in vaccinated animals. In animals subjected to IN vaccination, occasional mild hyperplasia of the bronchiolar lining epithelium could also be observed, which is considered a physiological response inherent to the route of administration rather than an adverse effect. Pathology scores remained low across all experimental groups, indicating overall minimal to mild tissue alterations. (**Supplementary Figures 3B, C**).

Following ID vaccination, under no infection conditions, most animals exhibited tissue scores ranging from 0 to 2, consistent with absent to mild focal changes (**Supplementary Figure 4A**). Upon infection, a slight increase in score variability was observed, with some values extending to scores of 3–4, indicating moderate multifocal involvement in a subset of samples; however, no statistically significant differences were detected between groups (*p* = 0.1851 and *p* = 0.5227). A similar pattern was observed following IN vaccination (**Supplementary Figure 4B**). In the absence of infection, scores were predominantly within the 0–2 range. Post-infection, most scores remained within the mild to moderate categories (scores 1–3), showing limited dispersion and no significant intergroup differences (*p* = 0.1491 and *p* = 0.0639), despite a slight trend towards higher scores in specific groups. Notably, a certain degree of heterogeneity was observed within and across groups, as reflected by the dispersion of individual scores.

Across both vaccination routes, partial scores indicated that the observed alterations were generally focal to multifocal and involved a limited proportion of the tissue (≤ 50%). The histologically identified inflammatory infiltrate was predominantly mononuclear, with a lymphoplasmacytic profile. This supports its interpretation as a mild, controlled immune response consistent with a chronic inflammatory model rather than severe or extensive tissue damage. Additionally, alveolar consolidation leading to reduced alveolar air spaces, along with focal reactivity of the visceral pleura, were occasional histopathological finding across samples. Analysis of myeloid populations (defined in **Supplementary Figure 5**) revealed no considerable changes attributable to either IN or ID vaccination. However, a significant decrease in the percentage of a myeloid Ly6C^−^ cell population, which may include interstitial macrophages and Ly6C^−^ monocytes, was observed in the lungs of infected animals that had been ID-vaccinated with the PAO1 ΔΔΔ strain, compared to the respective saline group (**Supplementary Figure 6**). A similar decrease relative to the saline controls was observed in this cell population in mice vaccinated IN with PAO1 ΔΔΔ Δ*htrB1* Δ*pagP* (**Supplementary Figure 7**).

#### 3.4.2 Protective efficacy in a murine model of chronic lung infection caused by *P. aeruginosa* DK1-NH57388A

To evaluate the protection conferred by ID and IN immunization with PAO1 ΔΔΔ or PAO1 ΔΔΔ Δ*htrB1* Δ*pagP*, we quantified lung bacterial burdens 13 days post-challenge with DK1-NH57388A. ID immunization with either strain resulted in a modest reduction of approximately 1.4-log units (*p* = 0.0659, Kruskal-Wallis test) (**Figure 3C**). In contrast, IN immunization with either strain significantly resulted in a pronounced reduction in pulmonary bacterial load of approximately 2-log-units compared with the saline control group (*p* = 0.0108, Kruskal-Wallis test and Dunn’s *post hoc* multiple comparisons test) (**Figure 3D**). Moreover, no significant difference in protective efficacy were observed between the two vaccine strains in this chronic infection model.

#### 3.4.3 Cellular immune response induced by vaccine strains in a murine model of chronic lung infection

**Figure 4:**
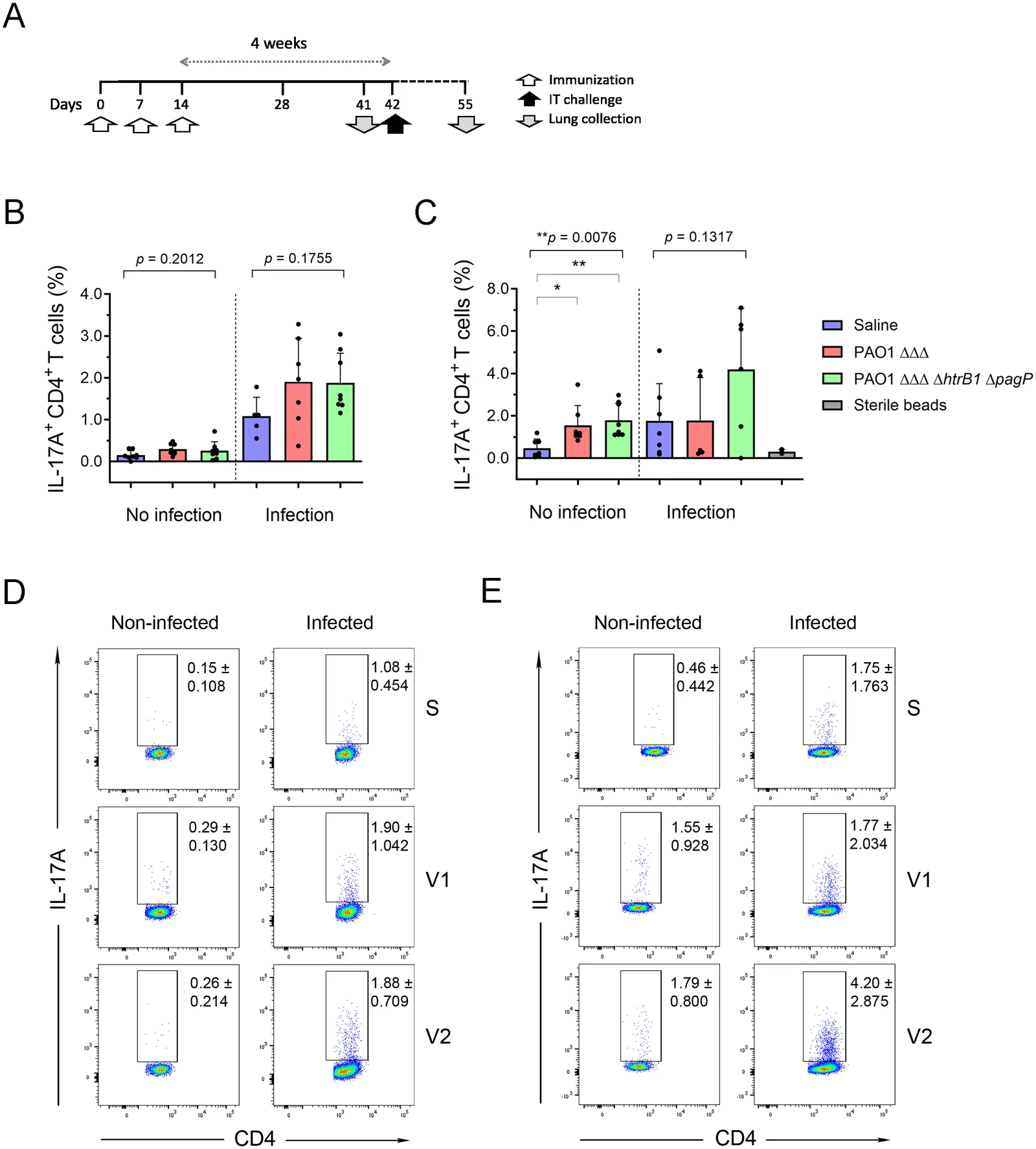
IL-17A production by lung CD4^+^ T cells from vaccinated mice. **(A)** Three-dose immunization schedule, challenge, and collection of lungs. **(B, C)** Mice (*n* = 16) were immunized with PAO1 ΔΔΔ or PAO1 ΔΔΔ Δ*htrB1* Δ*pagP* via the ID (1 × 10^8^ CFU) **(B)** or IN (2.5 × 10^7^ CFU) route **(C)**. Four weeks after the last vaccine dose, half of the mice in each group (*n* = 8) were challenged with DK1-NH57388A (1 × 10^6^ CFU) via the IT route. Control mice received sterile agar beads. Lungs were collected from vaccinated mice both before and after challenge. **(D, E)** Representative flow cytometry plots and frequencies of IL-17A^+^ CD4^+^ T cells in the lungs of ID- **(D)** or IN-immunized **(E)** mice before and after challenge. IL-17A was detected by intracellular cytokine staining after PMA/ionomycin stimulation. Gates were set using fluorescence minus one (FMO) and isotype control. Data are presented as mean ± SD, with individual mice shown as dots. Overall group differences were assessed using one-way ANOVA, followed by Tukey’s multiple-comparisons test (*p < 0.05, **p < 0.01). S; saline; V1, PAO1 ΔΔΔ; V2, PAO1 ΔΔΔ Δ*htrB1* Δ*pagP*.

**Figure 5:**
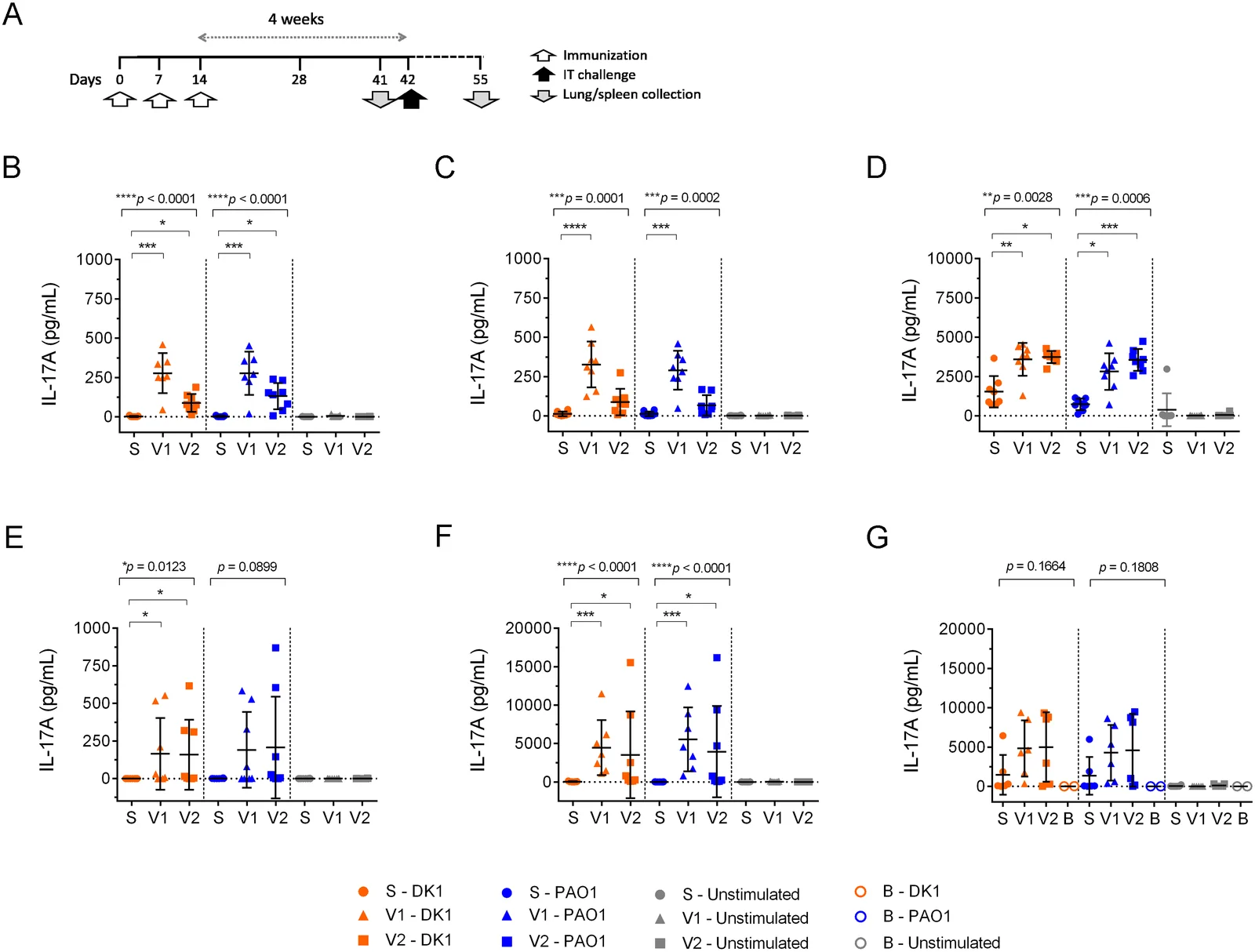
IL-17A secretion by CD4^+^ T cells from spleens and lungs of vaccinated mice. **(A)** Three-dose immunization schedule, challenge, and organ collection. **(B–G)** Mice (*n* = 16) were immunized with PAO1 ΔΔΔ or PAO1 ΔΔΔ Δ*htrB1* Δ*pagP* via the ID [1 × 10^8^ CFU; **(B–D)**] or IN [2.5 × 10^7^ CFU; **(E–G)**] route. Four weeks after immunization, half of the mice in each group (*n* = 8) were IT challenged with DK1-NH57388A embedded in agar beads (1 × 10^6^ CFU). Control mice received sterile beads. Spleens **(B, E)** and lungs **(C, D, F, G)** were collected 24 h before **(B, C, E, F)** and 13 days after challenge (**D, G**). IL-17A levels in splenocyte and lung cell cultures were measured by ELISA after 3-day stimulation with heat-killed (Hk) PAO1 (orange symbols) or Hk DK1-NH57388A (blue) at a 1:1 T cell to bacterium ratio. Unstimulated cells served as controls. Dots represent individual mice; horizontal lines indicate means; error bars, SD. Overall group differences were assessed using Kruskal-Wallis test, followed by Dunn’s *post hoc* multiple-comparisons test (*p < 0.05, **p < 0.01, ***p < 0.001, ****p < 0.0001). S; saline; V1, PAO1 ΔΔΔ; V2, PAO1 ΔΔΔ Δ*htrB1* Δ*pagP*; B, sterile beads; DK1, DK1-NH57388A.

To evaluate the cellular immune response elicited by the double auxotroph PAO1 ΔΔΔ and its LPS-modified derivative PAO1 ΔΔΔ Δ*htrB1* Δ*pagP*, single-cell suspensions were prepared from the spleens and lungs of ID- or IN-immunized mice (*n* = 8 per group), following the schedule shown in **Figures 4A** and **5A**. Samples were collected at baseline (pre-challenge) and 13 days post-challenge, and then analyzed by flow cytometry to characterize the CD4^+^ T cell cytokine profile (IL-17A, IFN-γ, IL-10, IL-4).

CD4^+^ T cell effector function was assessed by quantifying the proportions of cytokine-producing cells (gating strategy shown in **Supplementary Figure 8**). Following infection, ID-immunized mice exhibited a higher frequency of pulmonary IL-17A^+^ CD4^+^ T cells compared with saline controls (**Figures 4B, D**). In contrast, IN immunization with both strains induced a significant increase in pulmonary IL-17A^+^ CD4^+^ T cells prior to infection; however, post-infection, those vaccinated with PAO1 ΔΔΔ showed no difference relative to controls (**Figures 4C, E**) This was accompanied by greater variability among animals after challenge, which may have reduced the ability to detect differences between groups with the sample size used. Further analysis revealed a significant increase in IFN-γ^+^ and IL-10^+^ CD4^+^ T cells in ID-immunized mice (*p* < 0.001 and *p* < 0.01, respectively, Kruskal-Wallis test; **Supplementary Figures 9, 10**). By contrast, IFN-γ^+^ CD4^+^ T cells were reduced in the lungs of IN-vaccinated mice with PAO1 ΔΔΔ Δ*htrB1* Δ*pagP* compared with controls before infection (**Supplementary Figure 9**). No major differences in IL-4^+^ CD4^+^ T cell frequencies were detected (**Supplementary Figure 11**). Additionally, following specific *ex vivo* stimulation post-infection, splenic IL-17A- and IFN-γ-producing CD4^+^ T cell frequencies were significantly higher in the PAO1 ΔΔΔ ID-vaccinated group (**Supplementary Table 2**). Overall, these results indicate that the magnitude and functional profile of CD4^+^ T cell responses are dependent on both vaccination route and infection status.

To evaluate the immune response upon re-exposure to antigen, splenocytes and lung cells were isolated from mice immunized with PAO1 ΔΔΔ and PAO1 ΔΔΔ Δ*htrB1* Δ*pagP* before and after challenge (**Figure 5A**) and stimulated *ex vivo* with heat-inactivated *P. aeruginosa* PAO1 or DK1-NH57388A. Cytokine levels were measured by ELISA after 72 hours. IL-4 was excluded from further analyses due to inconsistent detection. In ID-vaccinated mice, stimulation triggered robust secretion of IL-17A (**Figures 5B–D**) and IFN-γ (**Supplementary Figures 12A–D**) in both spleen and lung cultures. While cytokine production was initially lower in the PAO1 ΔΔΔ Δ*htrB1* Δ*pagP* group compared with PAO1 ΔΔΔ (**Figures 5B, C**; **Supplementary Figures 12B, C**), post-infection lung samples showed comparable responses between the two strains (**Figure 5D**; **Supplementary Figure 12D**). Responses were independent of the bacterial stimulus used (PAO1 or DK1-NH57388A). IL-10 secretion was not significantly elevated in ID-vaccinated mice (**Supplementary Figures 13A–D**). In IN-immunized mice, lung cultures displayed elevated IL-17A (**Figures 5F, G**) and a modest increase in IFN-γ (**Supplementary Figures 12F–G**), though with high intragroup variability, which was also observed for IL-10 (**Supplementary Figures 13F–G**). Splenocytes from IN-vaccinated mice showed increased IL-17A (**Figure 5E**) and IL-10 (**Supplementary Figure 13E**), while IFN-γ levels (**Supplementary Figure 12E**) remained similar to controls. As an internal control, lung cells from mice receiving sterile agar beads via the IT route showed no detectable cytokine secretion (**Figure 5G**; **Supplementary Figures 12G, 13G**).

## 4 Discussion

In this study, we demonstrated that immunization with PAO1 Δ*murI* (and its derivative mutants) protects C57BL/6 mice against acute pneumonia and contributes to protection against chronic lung infection caused by *P. aeruginosa*, highlighting its potential as a broadly protective vaccine candidate. Previous studies have shown that several auxotrophic candidates protected BALB/c mice against acute lung infection caused by the hypervirulent strain PA14, achieving survival rates exceeding 90% (24, 25, 28, 36). In contrast, Priebe et al. reported that immunization of C3H/HeN mice with PAO1 Δ*aroA* failed to provide protection against PA14, resulting in 100% mortality (37, 38). These differences likely arise from variations in infection dose, mouse genetic background, or the intracellular tropism of the PAO1 Δ*aroA* vaccine strain, which may limit immune stimulation. Notably, immune responses to *P. aeruginosa* vary among inbred mouse strains: C3H/HeN and C57BL/6 typically exhibit Th1-biased responses, whereas BALB/c mice tend toward a predominantly Th2-type response (39–41).

During acute lung infection, *P. aeruginosa* is generally cleared through opsonizing antibodies and the recruitment of macrophages and neutrophils. Activated macrophages release inflammatory mediators that promote immune cell infiltration; however, excessive pro-inflammatory cytokine production can lead to immune-mediated damage of respiratory tissues. Under certain conditions, such as in CF, *P. aeruginosa* can evade the immune response to establish a persistent infection (5). Although CF patients initially develop an acute immune response, the antibodies generated during this phase often fail to control disease progression, raising questions about their effectiveness (42). Elevated specific IgG levels have been detected in patients with chronic *P. aeruginosa* infection; however, their association with clinical improvement remains unclear (43). Similarly, high IgA levels may limit bacterial dissemination without achieving full eradication of the pathogen from the lung (44). This does not diminish the importance of IgA, as mice deficient in the polymeric immunoglobulin receptor (pIgR), which is responsible for IgA transport, exhibit increased mortality following IT pulmonary infection (45).

Increasing evidence indicates that T cell responses, particularly those mediated by CD4^+^ T cells, play a critical role in shaping the cytokine profile associated with chronic *P. aeruginosa* infection and chronic lung disease more broadly (46). Clinical studies have revealed that CF patients with persistent *P. aeruginosa* infection exhibit a Th2-dominant response, whereas those without chronic infection display a Th1-type profile associated with elevated IFN-γ levels and improved lung function (47). Furthermore, studies in mice have shown that resistance to *P. aeruginosa* reinfection correlates with a Th1-skewed response and an increased IFN-γ/IL-4 ratio (48). The Th17 pathway also contributes to protection, as IL-17A promotes neutrophil recruitment, which is essential for controlling acute infection (38, 49). Indeed, neutralization of IL-17 or loss of its receptor reduces immune efficacy against acute pneumonia (38). Although the optimal immune profile remains uncertain, evidence suggests that mixed Th1/Th2 or Th1/Th17 responses confer protection, whereas a predominantly Th2-biased response appears is often suboptimal (49). Tissue-resident Th17 cells, generated by prior mucosal exposure or IN immunization, confer protective memory against a range of respiratory pathogens (50–52). In acute infection models, the protection conferred by PAO1 Δ*aroA* was abolished following IL-17 neutralization or in IL-17 receptor-deficient mice (38), a protective role further supported by other studies (28, 53–56). Notably, IN immunization of BALB/c mice with PAO1 Δ*murI* induced an increase in IL-17A-producing CD4^+^ T cells in the spleen and lungs, a finding further supported by elevated IL-17A levels in splenocyte culture supernatants stimulated with heat-inactivated PA14 (28). Protection against *P. aeruginosa* has also been associated with increased pulmonary production of IFN-γ and IL-10 (53); IFN-γ correlates with improved lung function (57), whereas elevated IL-10 levels are associated with reduced tissue damage during infection (58).

To optimize the chronic infection model, *P. aeruginosa* strains isolated from CF patients were selected, including the Danish strain DK1-NH57388A, the Liverpool Epidemic Strain LES400, and local isolates A and E from our hospital. Precise control of the IT inoculum was critical, as doses below 1 **×** 10^6^ CFU in C57BL/6 mice typically result in infection clearance. In our experiments, an optimal inoculum of 1 **×** 10^6^ CFU was established, consistent with previous studies identifying a range of 10^6^−10^7^ CFU as necessary to achieve chronic colonization (27). Due to strain-dependent differences in susceptibility, C57BL/6 mice were selected for their predisposition to develop chronic infection (27). Overall, the data suggest a potential dose-dependent effect of the bacterial inoculum on disease outcomes, whereas host factors showed weaker associations. However, these findings should be interpretated with caution, as the small sample size likely reduced statistical power, preventing the detection of statistically significant effects.

The selection of vaccine candidates for the chronic infection model was based on previously established criteria, including stimulation of the TLR4/MD-2 complex, attenuation and safety profiles, protective efficacy, and the capacity to induce antibody responses (25). Bioactivity assays of the mouse TLR4 (mTLR4)/MD2 complex revealed lower activation for the PAO1 ΔΔΔ Δ*htrB1* Δ*pagP* candidate compared to PAO1 ΔΔΔ Δ*htrB2* Δ*pagP*. Moreover, although PAO1 ΔΔΔ Δ*htrB1*, PAO1 ΔΔΔ Δ*htrB2* and PAO1 ΔΔΔ Δ*pagP* exhibited comparable attenuation following high-dose IP administration, PAO1 ΔΔΔ Δ*htrB1* was safer than PAO1 ΔΔΔ Δ*htrB2* when administered via the IN route. In terms of efficacy, immunization with PAO1 ΔΔΔ Δ*htrB1* resulted in slightly higher survival rates following PA14 challenge compared with PAO1 ΔΔΔ Δ*htrB2*, although the difference was not statistically significant, while eliciting comparable antibody responses (25). Based on these findings, PAO1 ΔΔΔ Δ*htrB1* Δ*pagP* was selected for evaluation in the chronic infection model alongside its parental strain PAO1 ΔΔΔ. In our study, vaccination with the tested strains resulted in a transient and mild reduction in body weight, which resolved rapidly and was not associated with progressive disease or mortality. Although the biological significance of this finding remains to be fully elucidated, body weight loss is a recognized indicator of respiratory distress in rodents. Consequently, this transient decrease may reflect a mild reactogenic response that warrants further investigation. ID administration was additionally associated with small, localized and self-limiting skin lesions at the injection site, which resolved spontaneously without treatment or systemic adverse effects, as previously reported in BALB/c mice (28). Consistent with this, histopathological analysis of skin from ID vaccinated mice at the time of infection confirmed normal epidermal and dermal architecture, indicating complete tissue recovery. Overall, these findings suggest a transient local reactogenicity profile. However, the potential implications for clinical acceptability should be considered in future development, particularly with respect to tolerability and vaccine uptake in the target population.

The enhanced protection observed following IN administration compared to the ID delivery raises intriguing questions regarding the underlying immunological mechanisms. One possibility is that mucosal administration of PAO1 auxotrophic strains might trigger localized trained immunity, providing an early innate defense that shapes subsequent immune responses. However, this early innate activation is likely complemented by the development of a robust adaptive memory response. Indeed, as shown in our previous work (28) with the PAO1 Δ*murI* strain, immunization elicited a characteristic adaptive immune signature. Specifically, the proportions of CD4^+^ T cells displaying CD44^+^ CD62L^−^ and CD44^+^ CD62L^+^ phenotypes were significantly increased in vaccinated mice compared to controls, suggesting the stimulation of both effector memory and central memory CD4^+^ T cells. Therefore, while local trained immunity may contribute to immediate mucosal reinforcement, the protective efficacy of the PAO1 Δ*murI* platform is likely reinforced by a well-established adaptive immune memory network.

Immunization with both strains increased the frequency of IL-17A-producing CD4^+^ T cells in the lungs. Similarly, IL-17A and IFN-γ production was significantly increased in splenocytes and lung cell cultures from ID-immunized mice. While cytokine levels were lower for the PAO1 ΔΔΔ Δ*htrB1* Δ*pagP* variant, likely due to reduced mTLR4 activation and lower endotoxicity, IN administration contributed to protection mice against chronic infection, reducing bacterial load. These results suggest that PAO1 ΔΔΔ Δ*htrB1* Δ*pagP* is a promising vaccine candidate, offering reduced LPS-associated reactivity without compromising the protective capacity of the parental strain.

The role of IL-17A in chronic infection is less defined than in acute models. However, IL-17A levels remain elevated in both mice and patients during chronic *P. aeruginosa* infection, coinciding with increased markers of tissue damage (59, 60). In this study, IFN-γ production in the lungs increased moderately following infection, whereas IL-17A production by CD4^+^ T cells was more pronounced. These findings are consistent with observations from nasal infection models of *B. pertussis*, in which antigen-specific Th17 cells secreting IL-17 are present at higher proportions than Th1 cells producing IFN-γ and elicit responses in subsequent infections (52, 61). The role of IL-17A during chronic *P. aeruginosa* infection appears to be complex, encompassing both protective and potentially pathological effects. While IL-17A promotes neutrophil recruitment and contributes to bacterial control, sustained activation of the IL-17A/IL-17RA axis has also been associated with increased pulmonary inflammation, extracellular matrix degradation, and tissue damage in experimental models of chronic infection as well as in patients with CF. Lorè et al. (2016) reported that genetic deficiency of IL-17A signaling increased bacterial persistence while simultaneously reducing markers of tissue injury, suggesting a trade-off between host resistance and host tolerance (60). Although IL-17A is typically linked to neutrophil recruitment, no differences in neutrophil proportions were observed in this study by flow cytometry or within lung tissue sections. Histologically, the inflammatory infiltrate was predominantly mononuclear with a lymphoplasmacytic profile, consistent with a chronic inflammatory response characterized by a mild, controlled immune reaction rather than extensive tissue damage. In this context, although the elevated IL-17A responses observed in vaccinated mice were associated with lower pulmonary bacterial burdens, their contribution to long-term lung pathology remains uncertain and warrants further investigation.

While several vaccine candidates against *P. aeruginosa* have been developed using skin and acute lung infection models, few have been tested in chronic infection models (62–64). Gilleland et al. (1993) demonstrated that rats immunized IM with OprF protein, elastase, and ETA exhibited less severe lesions following IT infection with a clinical isolate in agar particles (62). Similarly, Price et al. (2001) reported protective effects using a DNA vaccine encoding the *oprF* gene in mice (63), and Sokol et al. (2000) demonstrated reduced tissue damage and leukocyte infiltration in rats immunized with elastase conjugated with tetanus toxoid (64). Notably, most other candidates have failed to achieve a reduction in pulmonary bacterial load in chronic infection models. The interpretation of results in these models is further complicated by their inherent stringency and by the strong influence of experimental variables, including the bacterial strain, inoculation dose, and infection methodology, all of which can substantially affect infection establishment and persistence (26). Within this context, the reduced pulmonary bacterial burden and the lower proportion of colonized animals observed in vaccinated mice provide evidence of the beneficial effects of vaccination. Although protection was less pronounced than that observed in the acute challenge model, these findings suggest that vaccination with these auxotrophic strains can limit the establishment and persistence of chronic lung infection. While histopathological analyses were performed, more comprehensive studies will be required to fully characterize the effects of vaccination on lung tissue architecture, inflammation, and the potential remodeling associated with chronic infection. Therefore, despite the need for further safety and mechanistic studies to fully refine its profile, PAO1 ΔΔΔ Δ*htrB1* Δ*pagP* represents a promising vaccine candidate that offers a favorable balance between protective efficacy and limited reactogenicity compared with previously reported approaches.

## Data Availability

The datasets presented in this study can be found in online repositories. The names of the repository/repositories and accession number(s) can be found in the article/**Supplementary Material**.
