# Supplementary material for: Live auxotrophic vaccines contribute to protection against chronic lung infections caused by Pseudomonas aeruginosa in C57BL/6 mice
Source: Front Immunol. 2026 Sep 3;17:1862061. doi: 10.3389/fimmu.2026.1862061 (PMC13582316; doi:10.3389/fimmu.2026.1862061)
Supplement: Supplementary file 1 [file Presentation1.pdf]

Supplementary Material

Live auxotrophic vaccines contribute to protection against chronic lung infections caused by *Pseudomonas aeruginosa* in C57BL/6 mice

Víctor Fuentes-Valverde[1,2†‡], Alexandra Correia[3,4‡], Patricia García[1,2], Elena Pérez-Anton[3], Irina Amorim[3,5], Soraya Rumbo-Feal[1], Maria P. Cabral[1†], Manuel Vilanova[3,4], Miriam Moscoso[1,2*], Germán Bou[1,2,6*]

[1]Servicio de Microbiología Clínica and Grupo de Investigación en Microbiología, Instituto de Investigación Biomédica de A Coruña (INIBIC), Complexo Hospitalario Universitario de A Coruña (CHUAC), Servizo Galego de Saúde (SERGAS), Universidade da Coruña, A Coruña, Spain. [2]Centro de Investigación Biomédica en Red de Enfermedades Infecciosas (CIBERINFEC), Instituto de Salud Carlos III, Madrid, Spain. [3]i3S Instituto de Investigação e Inovação em Saúde, Universidade do Porto, Porto, Portugal. [4]Departamento de Imuno-Fisiologia e Farmacologia, Instituto de Ciências Biomédicas Abel Salazar, Universidade do Porto, Porto, Portugal. [5]Departamento de Patologia e Imunologia Molecular, Instituto de Ciências Biomédicas Abel Salazar, Universidade do Porto, Porto, Portugal. [6]Departamento de Fisioterapia, Medicina e Ciencias Biomédicas, Universidade da Coruña, A Coruña, Spain.

[†]Present address: V.F-V., Área de Medicamentos Biológicos, Agencia Española de Medicamentos y Productos Sanitarios (AEMPS), Madrid, Spain; M.P.C., CZ Vaccines SAU, O Porriño, Spain.

*Correspondence: M.M., mirian.moscoso.naya@sergas.es, G.B., german.bou.arevalo@sergas.es

[‡]V.F-V. and A.C. contributed equally to this work.

1 Supplementary Figures and Tables

1.1 Supplementary Figures

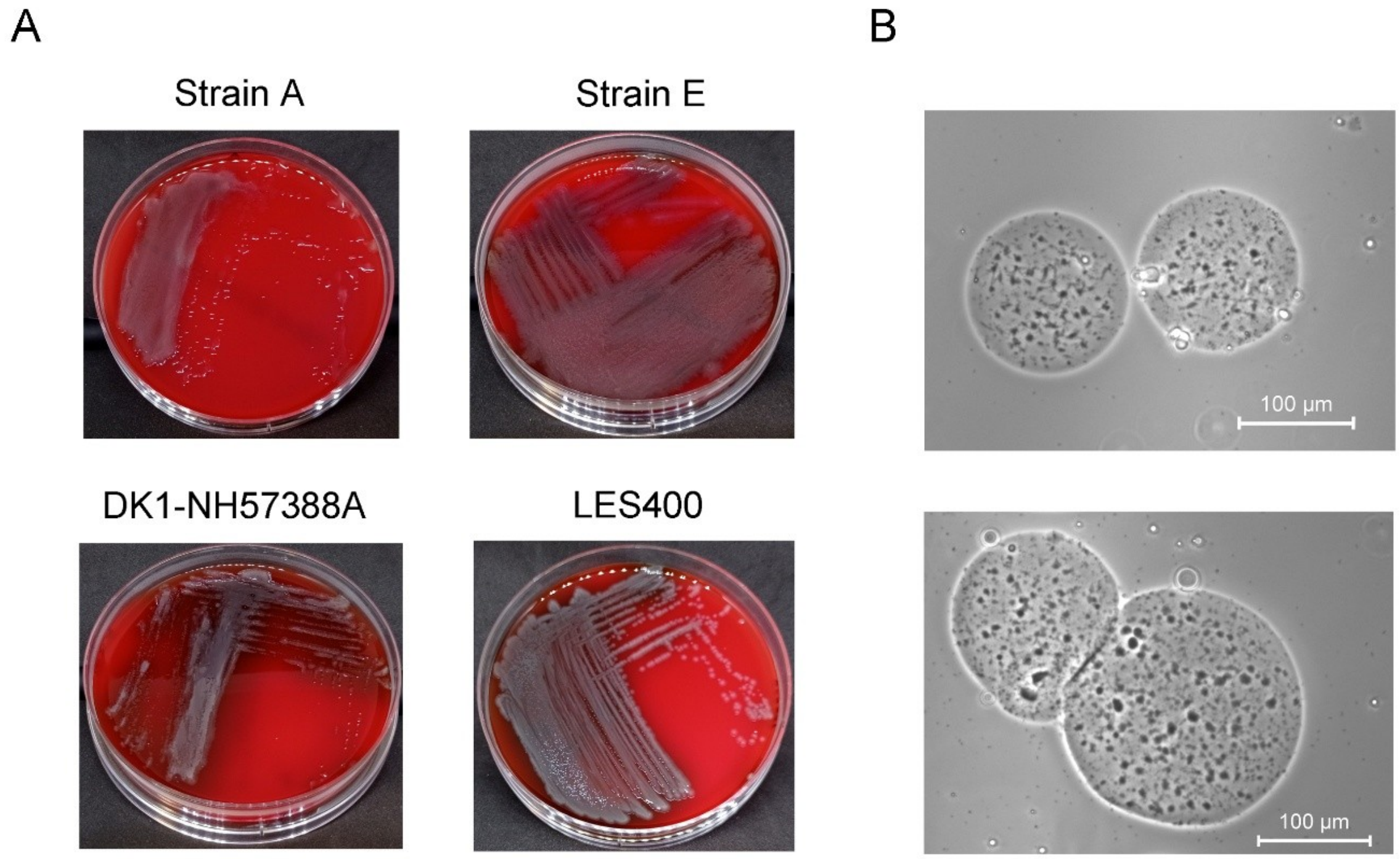

Supplementary Figure 1. (**A**) Selected mucoid *P. aeruginosa* strains: strain A (CF-51419182), strain E (CF-51429475), DK1-NH57388A and LES400 grown on TSA-S plates at 37ºC for 48 h. (**B**) DK1-NH57388A embedded in agar beads. Images acquired 24 h after bead preparation using a Nikon Eclipse Ti inverted fluorescence microscope at 10× magnification.

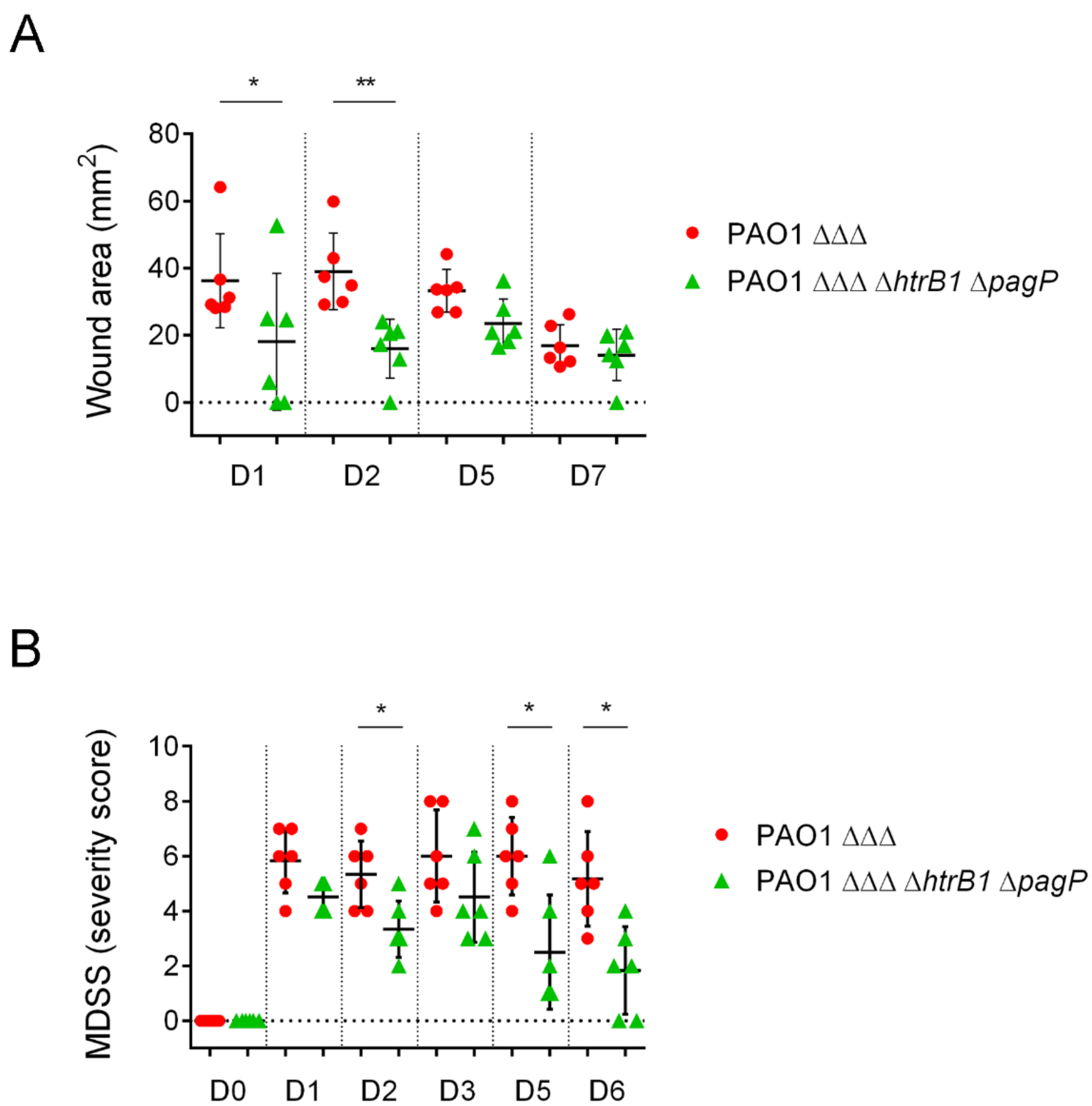

Supplementary Figure 2. Local and systemic reactogenicity following immunization with vaccine candidates. (**A**) Wound area (mm^2) measurements of skin lesions observed in C57BL/6 mice ($n = 6$ per group) following intradermal (ID) immunization with vaccine candidates PAO1 ΔΔΔ or PAO1 ΔΔΔ Δ*htrB1* Δ*pagP* (1×10^8 CFU). (**B**) Murine disease severity score (MDSS) following intranasal (IN) administration of high-dose vaccine candidates (8×10^8 CFU) in C57BL/6 mice ($n = 6$ per group). Data are shown as individual values per mouse. *$p < 0.05$, **$p < 0.01$, Mann-Whitney U test.

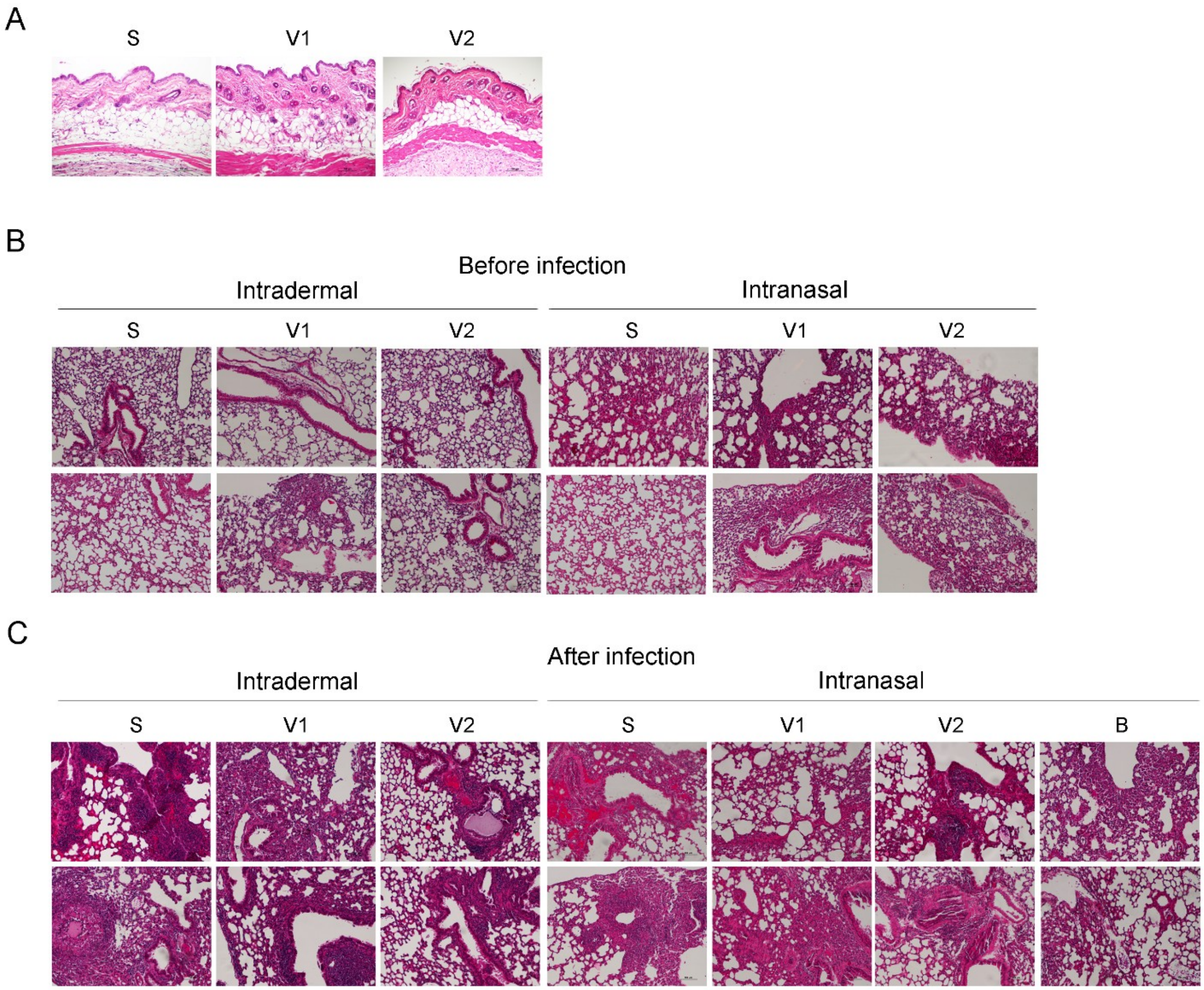

Supplementary Figure 3. Skin and lung histopathology following chronic respiratory infection with *P. aeruginosa* in vaccinated and unvaccinated mice. Mice (n = 16) were immunized with PAO1 ΔΔΔ or PAO1 ΔΔΔ Δ*htrB1* Δ*pagP* via the ID (1×10^8 CFU) or IN (2.5×10^7 CFU) route. Four weeks after the final immunization, half of the mice in each group (n = 8) were challenged with DK1-NH57388A (1×10^6 CFU) via the IT route. Control mice received sterile agar beads. (**A**) Representative histological sections of skin (H&E staining, ×100 magnification, scale bar = 100 µm) from ID immunized mice at the time of infection. The overall skin architecture was preserved across all groups, with normal stratification of the epidermis and dermis. Hair follicles were evident and present at various stages of development. Histopathological examination revealed no relevant alterations, and no significant differences were observed among the experimental groups. (**B, C**) Representative lung histology sections collected before and after infection from animals following ID or IN administration (H&E, ×100). Before infection (**B**), H&E-stained lung sections showed an overall preserved pulmonary architecture, with occasional infiltration or major histopathological alterations. After infection (**C**), H&E-stained lung sections revealed lesions of variable severity, including multifocal mononuclear inflammatory infiltrates, alveolar septal thickening, peribronchiolar inflammation, and focal areas of pulmonary consolidation. The severity and distribution of histopathological alterations varied among treatment groups and routes of administration. S, saline; V1, PAO1 ΔΔΔ; V2, PAO1 ΔΔΔ Δ*htrB1* Δ*pagP*; B, sterile beads.

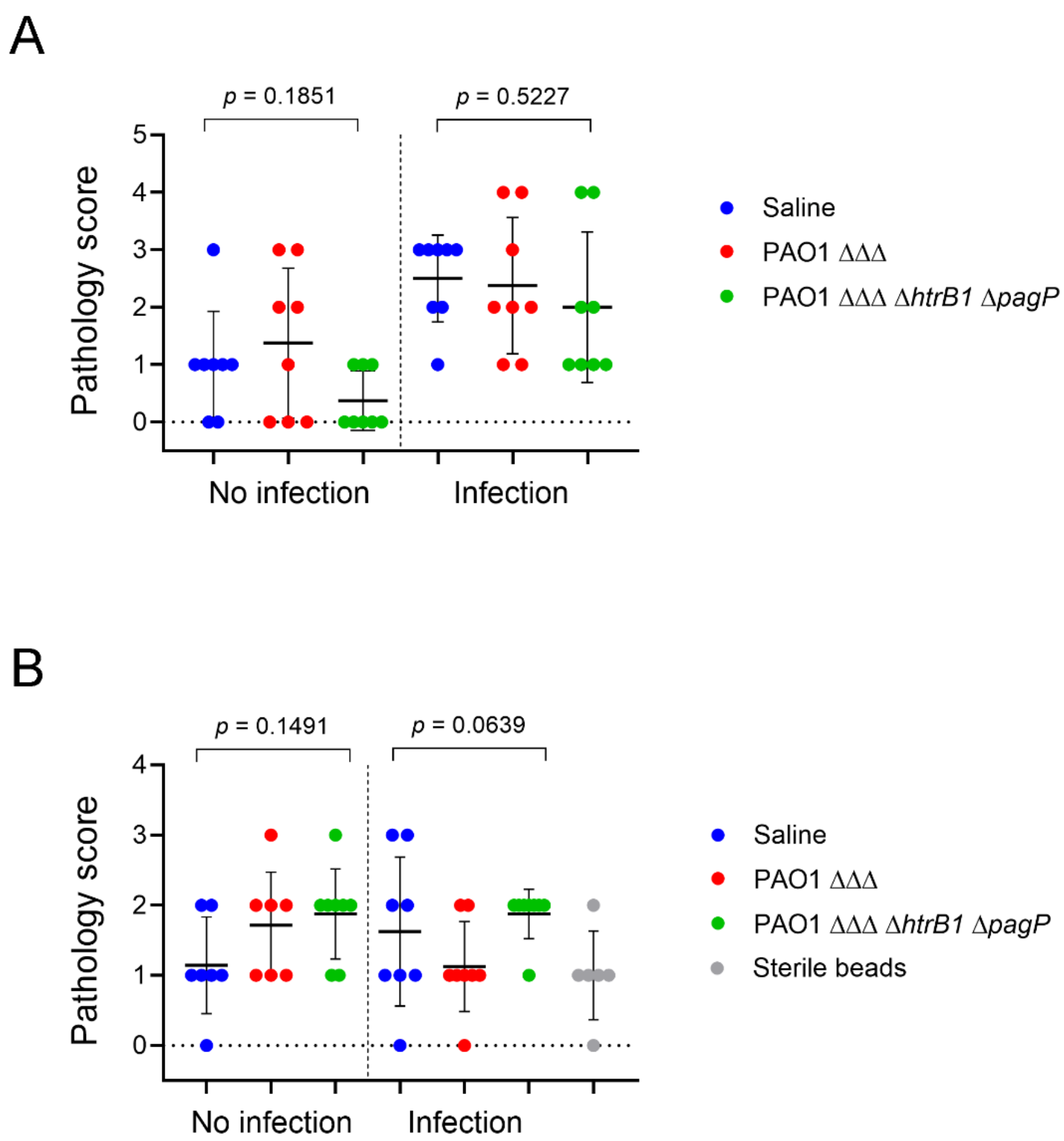

Supplementary Figure 4. Histopathological scores of lung sections from vaccinated mice. Mice (n = 16) were immunized with PAO1 ΔΔΔ or PAO1 ΔΔΔ Δ*htrB1* Δ*pagP* via the ID (1×10^8 CFU) (**A**) or IN (2.5×10^7 CFU) route (**B**). Four weeks after the final vaccine dose, half of the mice in each group (n = 8) were challenged with DK1-NH57388A (1×10^6 CFU) via the IT route. Control mice received sterile agar beads. Lungs were collected from vaccinated mice either before challenge or 13 days post-challenge. Data are presented as mean ± SD, with individual mice shown as dots. Each dot represents the histopathology score assigned to a lung section: 0, no lesions/alterations; 1, minimal alterations; 2, mild alterations; 3, moderate alterations; 4, moderate to severe alterations; and 5, marked (severe) alterations. p-value, according Kruskal-Wallis test with Dunn's *post hoc* test.

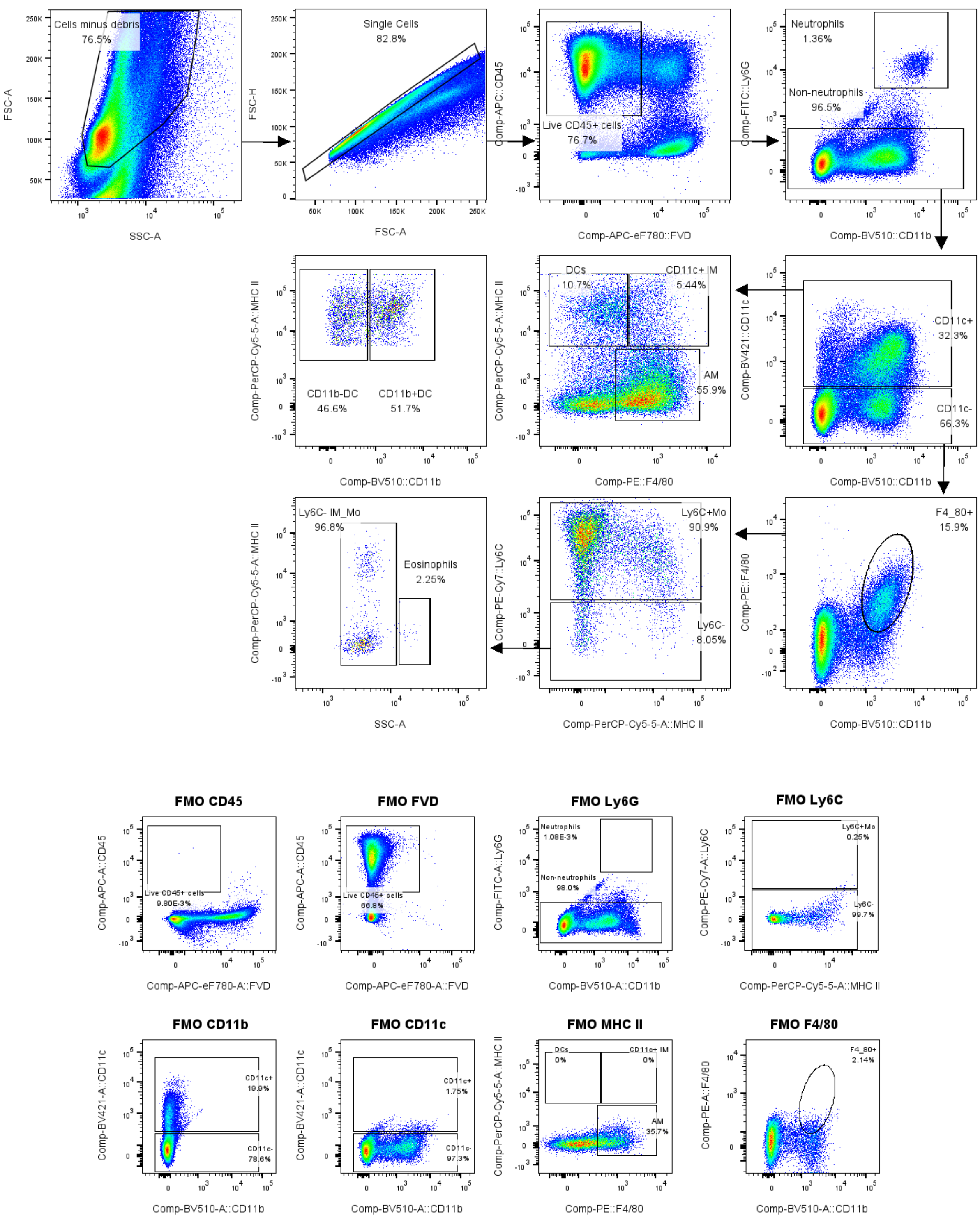
Cells minus debris
76.5%
FSC-A
SSC-A
Single Cells
82.8%
FSC-H
FSC-A
Comp-APC::CD45
Live CD45+ cells
76.7%
Comp-APC-eF780::FVD
Neutrophils
1.36%
Non-neutrophils
96.5%
Comp-FITC::Ly6G
Comp-BV510::CD11b
CD11c+
32.3%
CD11c-
66.3%
Comp-BV421::CD11c
Comp-BV510::CD11b
DCs
10.7%
CD11c+ IM
5.44%
AM
55.9%
Comp-PerCP-Cy5-5-A::MHC II
Comp-PE::F4/80
CD11b-DC
46.6%
CD11b+DC
51.7%
Comp-PerCP-Cy5-5-A::MHC II
Comp-BV510::CD11b
F4_80+
15.9%
Comp-PE::F4/80
Comp-BV510::CD11b
Ly6C+Mo
90.9%
Ly6C-
8.05%
Comp-PE-Cy7::Ly6C
Comp-PerCP-Cy5-5-A::MHC II
Ly6C- IM_Mo
96.8%
Eosinophils
2.25%
Comp-PerCP-Cy5-5-A::MHC II
SSC-A
FMO CD45
Live CD45+ cells
9.80E-3%
Comp-APC-A::CD45
Comp-APC-eF780-A::FVD
FMO FVD
Live CD45+ cells
66.8%
Comp-APC-A::CD45
Comp-APC-eF780-A::FVD
FMO Ly6G
Neutrophils
1.08E-3%
Non-neutrophils
98.0%
Comp-FITC-A::Ly6G
Comp-BV510-A::CD11b
FMO Ly6C
Ly6C+Mo
0.25%
Ly6C-
99.7%
Comp-PE-Cy7-A::Ly6C
Comp-PerCP-Cy5-5-A::MHC II
FMO CD11b
CD11c+
19.9%
CD11c-
78.6%
Comp-BV421-A::CD11c
Comp-BV510-A::CD11b
FMO CD11c
CD11c+
1.75%
CD11c-
97.3%
Comp-BV421-A::CD11c
Comp-BV510-A::CD11b
FMO MHC II
DCs
0%
CD11c+ IM
0%
AM
35.7%
Comp-PerCP-Cy5-5-A::MHC II
Comp-PE::F4/80
FMO F4/80
F4_80+
2.14%
Comp-PE-A::F4/80
Comp-BV510-A::CD11b

Supplementary Figure 5. Gating strategy used to identify myeloid cell populations and dendritic cells in the lungs of mice by flow cytometry. Cells were selected based on forward and side scatter characteristics (FSC and SSC, respectively) to exclude cellular debris; doublets were then excluded (single cells) and viable leukocytes were identified based on expression of the CD45 molecule and the absence of incorporation of the fixable viability dye (FVD); neutrophils were defined as $Ly6G^{+}CD11b^{+}$ cells and non-neutrophils were further divided in CD11c positive and negative cells; within $CD11c^{+}$, cells were identified as dendritic cells (DC), CD11c-expressing resident macrophages, namely a subtype of interstitial macrophages ($CD11c^{+}$ IM) and alveolar macrophages (AM), based on their expression of MHC class II and F4/80; DC were divided into CD11b positive and negative; within the $CD11c^{-}$ population, the F4/80-expressing cells were divided based on their Ly6C expression into $Ly6C^{+}$ monocytes ($Ly6C^{+}$ Mo) and a population containing interstitial macrophages and $Ly6C^{-}$ monocytes ($Ly6C^{-}$ IM_Mo). Eosinophils were defined as $Ly6C^{-}SSC^{hi}$. Representative pseudocolor dot plots of lung cells from a mouse from the saline group thirteen days after IT challenge with DK1-NH57388A embedded in agar beads. The different gates were defined using 'fluorescence minus one' (FMO) controls.

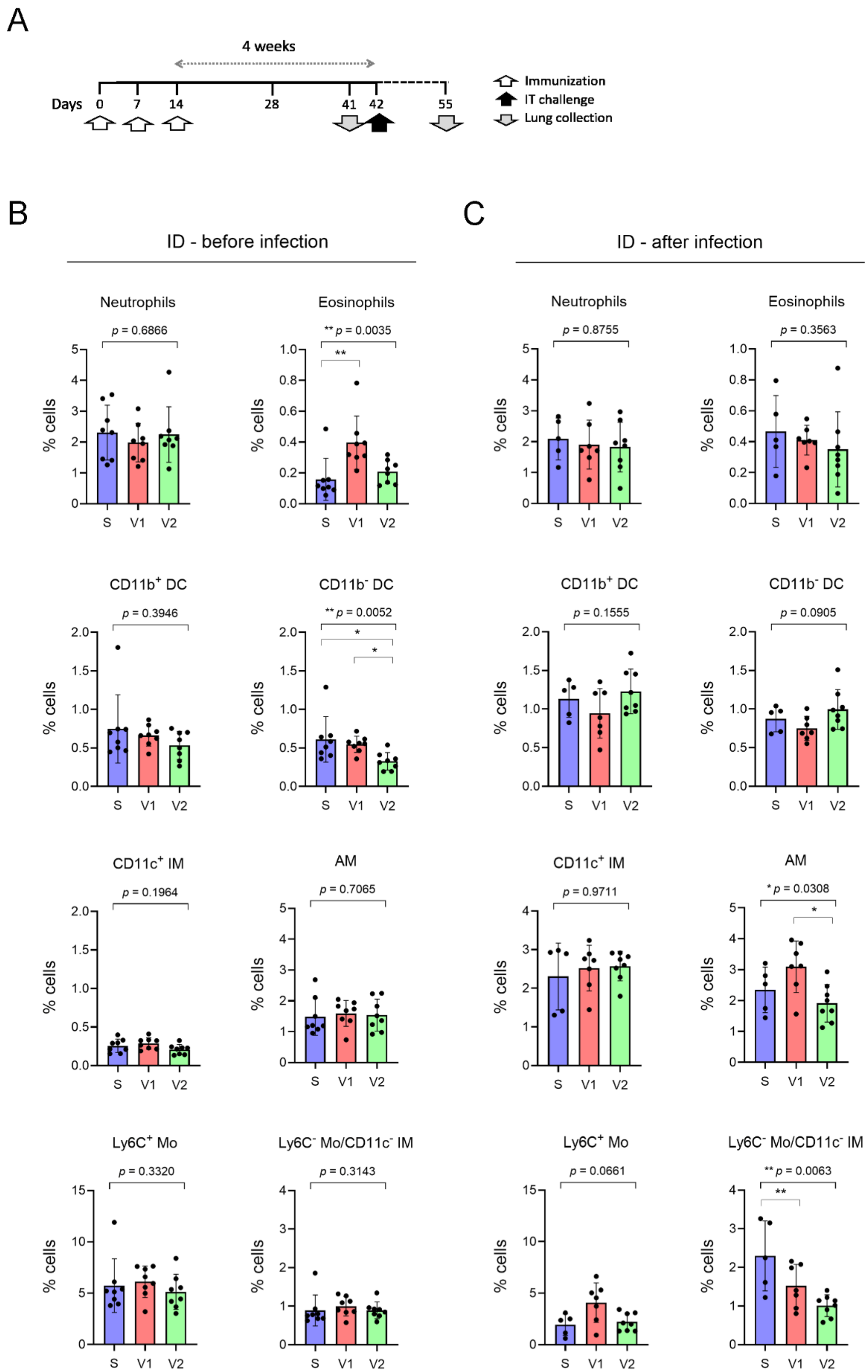
A
4 weeks
Days 0 7 14 28 41 42 55
Immunization
IT challenge
Lung collection
B
ID - before infection
C
ID - after infection
Neutrophils
p = 0.6866
Eosinophils
** p = 0.0035
**
Neutrophils
p = 0.8755
Eosinophils
p = 0.3563
CD11b+ DC
p = 0.3946
CD11b- DC
** p = 0.0052
*
*
CD11b+ DC
p = 0.1555
CD11b- DC
p = 0.0905
CD11c+ IM
p = 0.1964
AM
p = 0.7065
CD11c+ IM
p = 0.9711
AM
* p = 0.0308
*
Ly6C+ Mo
p = 0.3320
Ly6C- Mo/CD11c- IM
p = 0.3143
Ly6C+ Mo
p = 0.0661
Ly6C- Mo/CD11c- IM
** p = 0.0063
**
% cells
S V1 V2

Supplementary Figure 6. Frequency of myeloid cell populations (within $CD45^+$ live cells) in the lungs of control and ID vaccinated mice before and after challenge. (**A**) Three-dose immunization schedule, challenge, and lung collection time points. (**B, C**) Mice (n = 16) were immunized with PAO1 ΔΔΔ or PAO1 ΔΔΔ Δ*htrB1* Δ*pagP* via the ID (1×10^8 CFU). Four weeks after immunization, half of the mice in each group (n = 8) were challenged with DK1-NH57388A (1×10^6 CFU) via the IT route. Control mice received sterile beads. Lungs were collected from vaccinated mice both before (**B**) and after challenge (**C**). Bars show mean ± SD, and dots represent individual mice. *$p < 0.05$, **$p < 0.01$, Kruskal-Wallis test with Dunn's *post hoc* test. S; saline; V1, PAO1 ΔΔΔ; V2, PAO1 ΔΔΔ Δ*htrB1* Δ*pagP*.

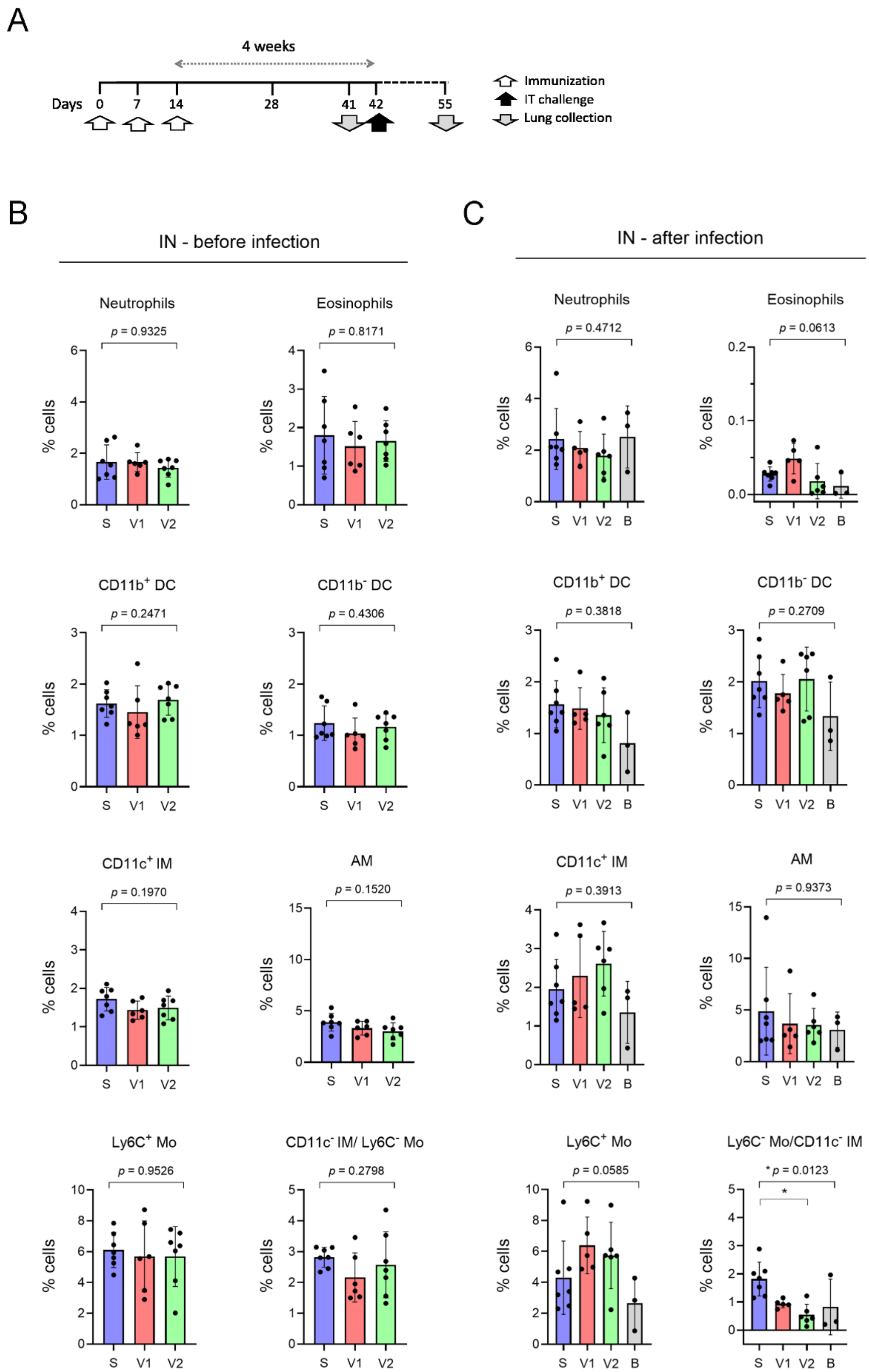
A
4 weeks
Days 0 7 14 28 41 42 55
Immunization
IT challenge
Lung collection
B
IN - before infection
C
IN - after infection
Neutrophils
p = 0.9325
Eosinophils
p = 0.8171
Neutrophils
p = 0.4712
Eosinophils
p = 0.0613
CD11b+ DC
p = 0.2471
CD11b- DC
p = 0.4306
CD11b+ DC
p = 0.3818
CD11b- DC
p = 0.2709
CD11c+ IM
p = 0.1970
AM
p = 0.1520
CD11c+ IM
p = 0.3913
AM
p = 0.9373
Ly6C+ Mo
p = 0.9526
CD11c- IM/ Ly6C- Mo
p = 0.2798
Ly6C+ Mo
p = 0.0585
Ly6C- Mo/CD11c- IM
* p = 0.0123
*
% cells
S V1 V2
S V1 V2 B

Supplementary Figure 7. Frequency of myeloid cell populations (within $CD45^+$ live cells) in the lungs of control and IN vaccinated mice before and after challenge. (**A**) Three-dose immunization schedule, challenge, and lung collection time points. Mice (n = 16) were immunized with PAO1 ΔΔΔ or PAO1 ΔΔΔ Δ*htrB1* Δ*pagP* via the IN (2.5×10^7 CFU) route (**C**). Four weeks after immunization, half of the mice in each group (n = 8) were challenged with DK1-NH57388A (1×10^6 CFU) via the IT route. Control mice received sterile beads. Lungs were collected from vaccinated mice both before (**B)** and after challenge (**C**). Bars show mean ± SD, and dots represent individual mice. *$p < 0.05$, Kruskal-Wallis test with Dunn's *post hoc* test. S; saline; V1, PAO1 ΔΔΔ; V2, PAO1 ΔΔΔ Δ*htrB1* Δ*pagP*; B, sterile beads.

Lungs

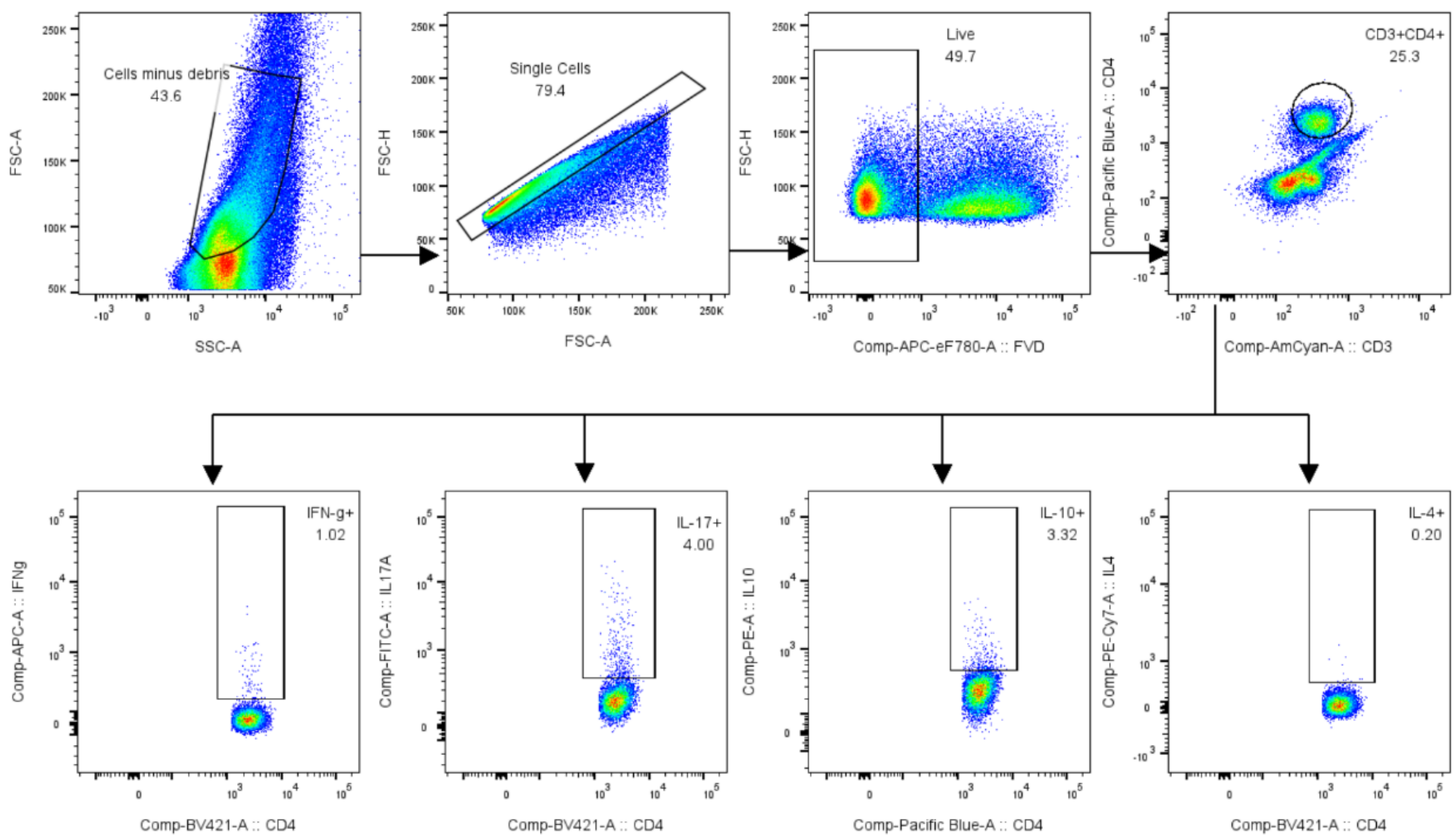

Spleen

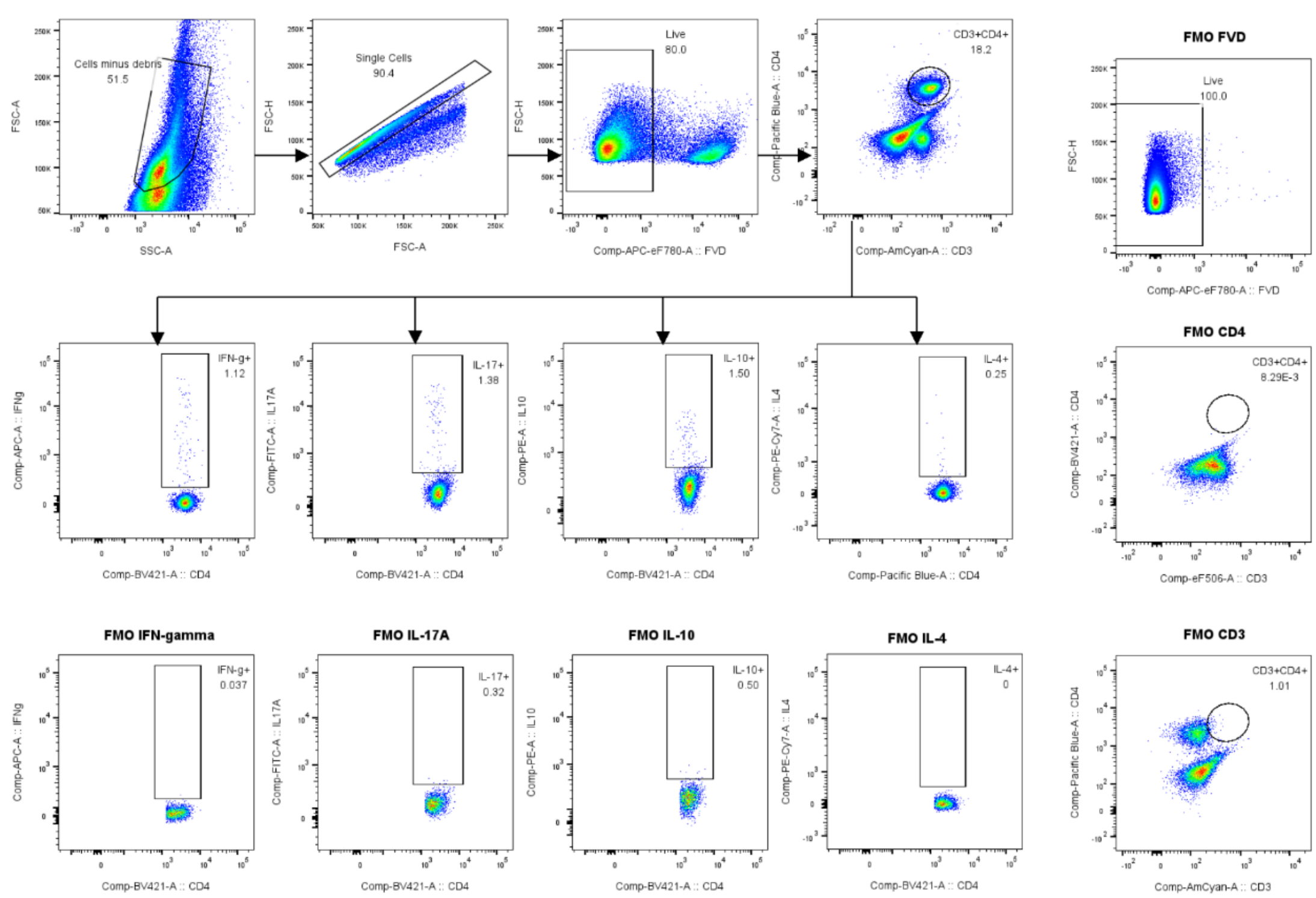

Supplementary Figure 8. Flow cytometry gating strategy for identifying cytokine-producing $CD4^+$ T cells in mouse lungs and spleen. Cells were selected based on forward and side scatter characteristics (FSC and SSC, respectively) to exclude debris. Doublets were excluded (single cells) and viable cells were identified using a fixable viability dye (FVD). $CD4^+$ T cells were defined as a subset of lymphocytes co-expressing CD3 and CD4 surface markers ($CD3^+$ $CD4^+$). Using fluorescence minus one (FMO) controls, gates on dot plots were set to distinguish cells producing IFN-γ, IL-17A, IL-10 and IL-4 from non-producing cells. Representative pseudocolor dot plots of lung and splenic $CD4^+$ T cells producing each cytokine are shown, along with the FMO controls.

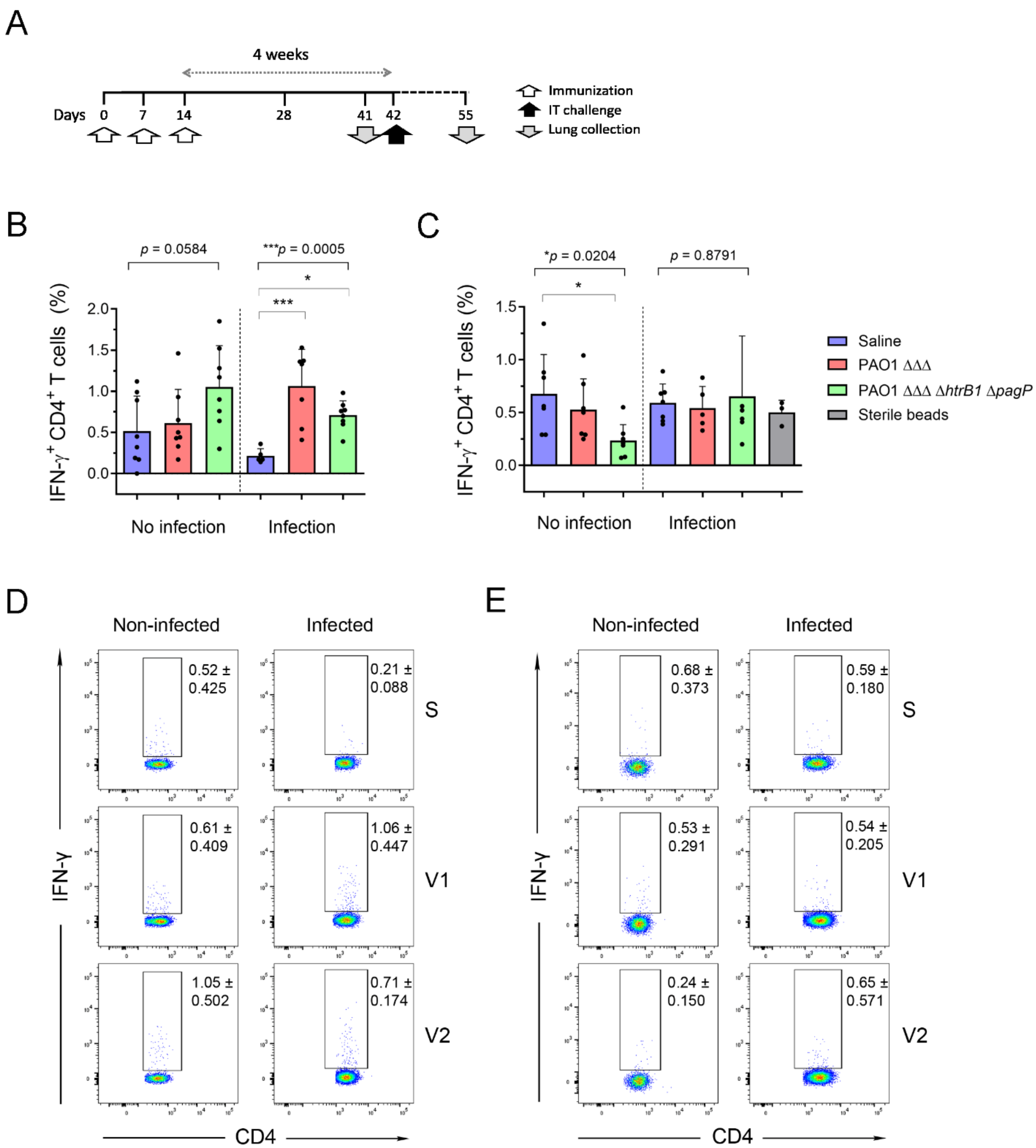

Supplementary Figure 9. IFN-γ production by lung CD4$^+$ T cells from vaccinated mice. (**A**) Three-dose immunization schedule, challenge, and lung collection time points. (**B, C**) Mice (n = 16) were immunized with PAO1 ΔΔΔ or PAO1 ΔΔΔ Δ*htrB1* Δ*pagP* via the ID (1 × 10^8 CFU) (**B**) or IN (2.5 × 10^7 CFU) route (**C**). Four weeks after immunization, half of the mice in each group (n = 8) were challenged with DK1-NH57388A (1 × 10^6 CFU) via the IT route. Control mice received sterile agar beads. Lungs were collected from vaccinated mice both before and after challenge. (**D, E**)

Representative flow cytometry plots and frequencies of IFN-γ^+ $CD4^+$ T cells in the lungs of ID- (**D**) or IN-immunized (**E**) mice before and after challenge. IFN-γ was detected by intracellular cytokine staining after PMA/ionomycin stimulation. Gates were set using fluorescence minus one (FMO) and isotype control. Data are presented as mean ± SD, with individual mice shown as dots. Overall group differences were assessed using one-way ANOVA, followed by Tukey's multiple-comparisons test (**p* < 0.05, ****p* < 0.001. S; saline; V1, PAO1 ΔΔΔ; V2, PAO1 ΔΔΔ Δ*htrB1* Δ*pagP*.

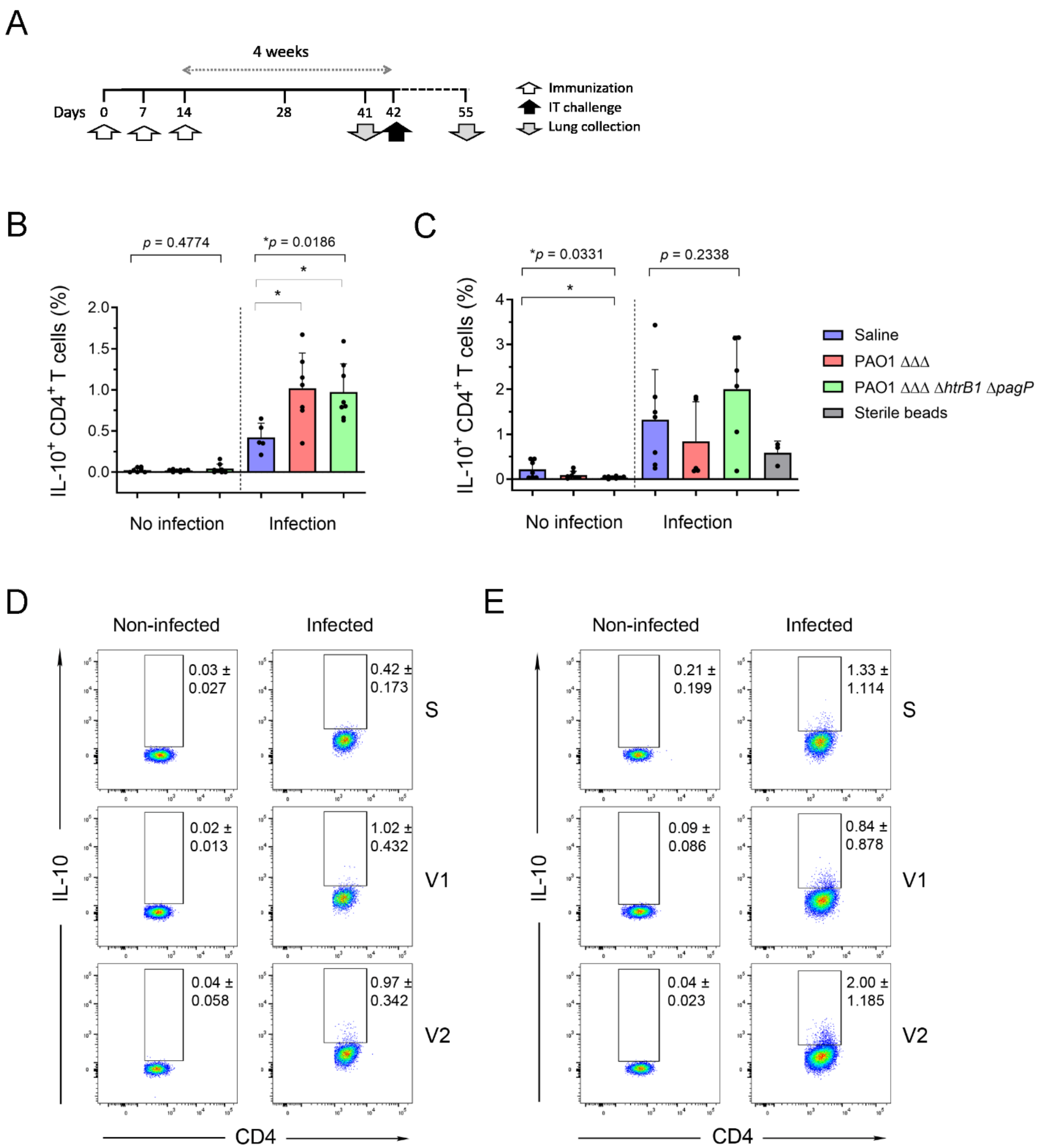

Supplementary Figure 10. IL-10 production by lung CD4$^+$ T cells from vaccinated mice. (**A**) Three-dose immunization schedule, challenge, and lung collection time points. (**B, C**) Mice (n = 16) were immunized with PAO1 ΔΔΔ or PAO1 ΔΔΔ Δ*htrB1* Δ*pagP* via the ID (1 × 10^8 CFU) (**B**) or IN (2.5 × 10^7 CFU) route (**C**). Four weeks after immunization, half of the mice in each group (n = 8) were challenged with DK1-NH57388A (1 × 10^6 CFU) via the IT route. Control mice received sterile beads. Lungs were collected from vaccinated mice both before and after challenge. (**D, E**) Representative

flow cytometry plots and frequencies of IL-10^+ $CD4^+$ T cells in the lungs of ID- (**D**) or IN-immunized (**E**) mice before and after challenge. IL-10 was detected by intracellular cytokine staining after PMA/ionomycin stimulation. Gates were set using FMO and isotype control. Data are presented as mean ± SD, with individual mice shown as dots. Overall group differences were assessed using one-way ANOVA, followed by Tukey's multiple-comparisons test (*$p < 0.05$). S; saline; V1, PAO1 ΔΔΔ; V2, PAO1 ΔΔΔ Δ*htrB1* Δ*pagP*.

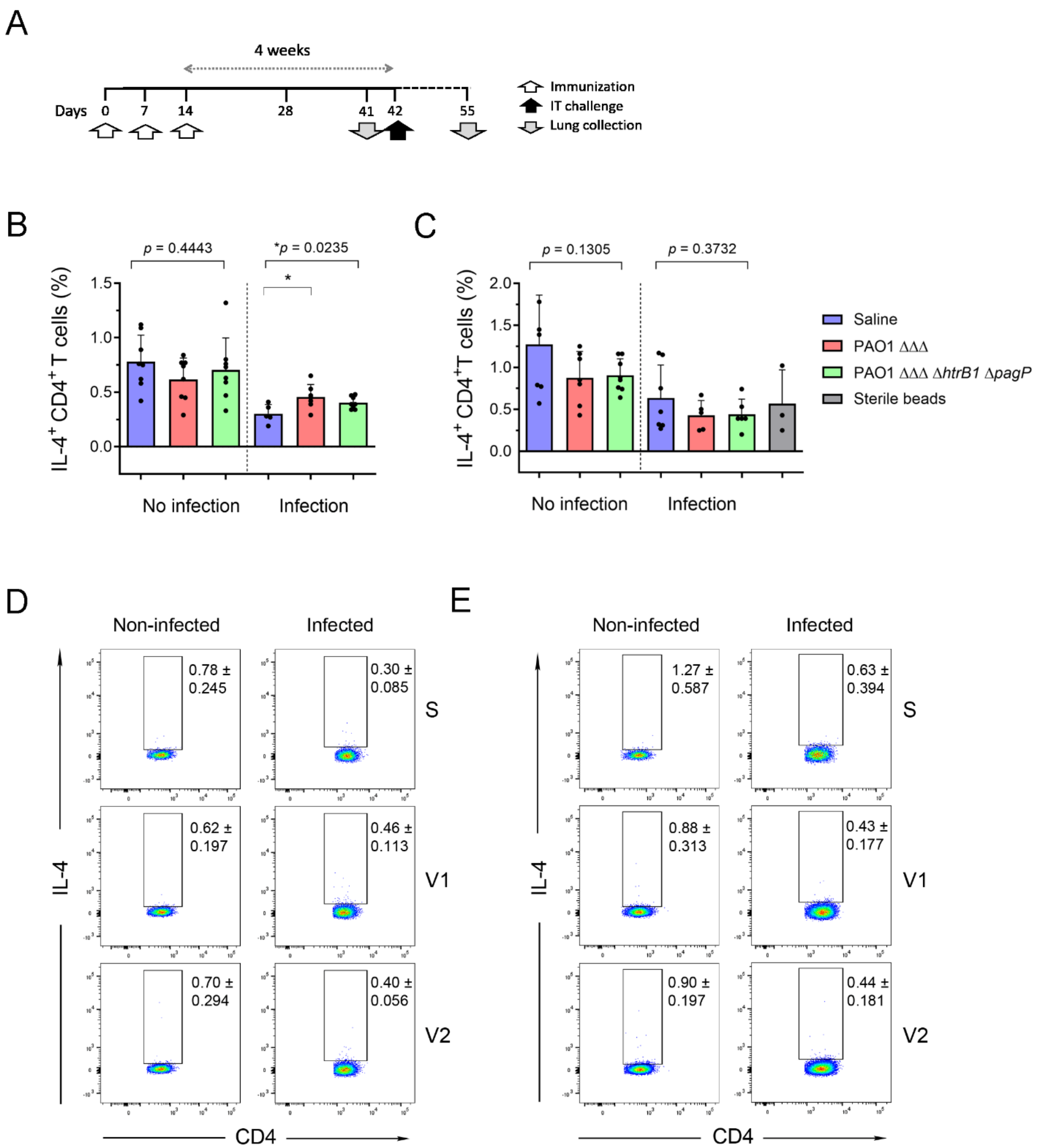

Supplementary Figure 11. IL-4 production by lung $CD4^+$ T cells from vaccinated mice. (**A**) Three-dose immunization schedule, challenge, and lung collection time points. (**B, C**) Mice (n = 16) were immunized with PAO1 ΔΔΔ or PAO1 ΔΔΔ Δ*htrB1* Δ*pagP* via the ID (1 × 10^8 CFU) (**B**) or IN (2.5 × 10^7 CFU) route (**C**). Four weeks after immunization, half of the mice in each group (n = 8) were challenged with DK1-NH57388A (1 × 10^6 CFU) via the IT route. Control mice received sterile beads. Lungs were collected from vaccinated mice both before and after challenge. (**D, E**) Representative

flow cytometry plots and frequencies of IL-4$^+$ CD4$^+$ T cells in the lungs of ID- (**D**) or IN-immunized (**E**) mice, before and after challenge. IL-10 was detected by intracellular cytokine staining after PMA/ionomycin stimulation. Gates were set using FMO and isotype control. Data are presented as mean ± SD, with individual mice shown as dots. Overall group differences were assessed using one-way ANOVA, followed by Tukey's multiple-comparisons test (*$p < 0.05$). S; saline; V1, PAO1 ΔΔΔ; V2, PAO1 ΔΔΔ Δ*htrB1* Δ*pagP*.

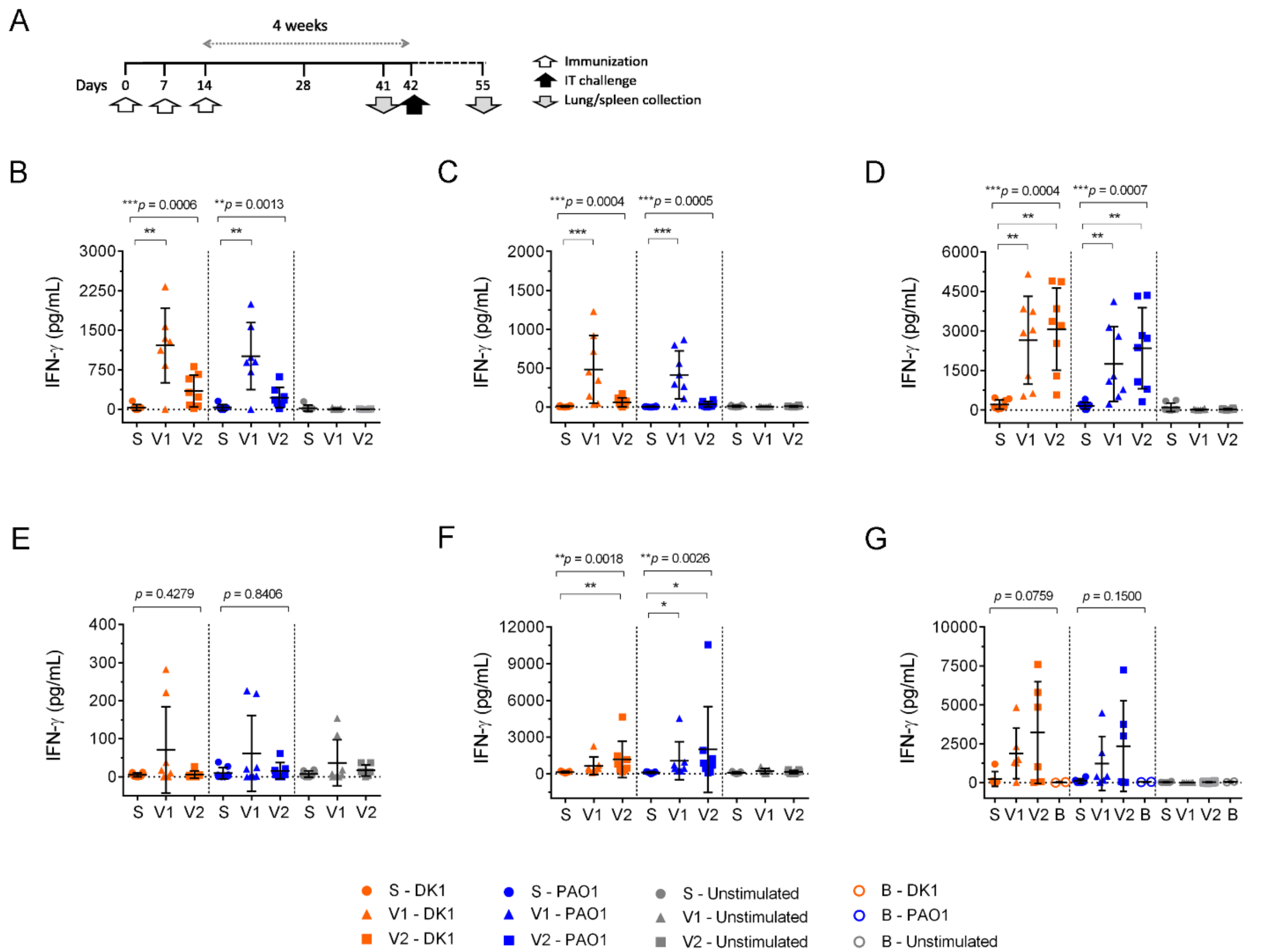

Supplementary Figure 12. IFN-γ secretion by $CD4^+$ T cells from spleens and lungs of vaccinated mice. (**A**) Three-dose immunization schedule, challenge, and organ collection. (**B-G**) Mice (n = 16) were immunized with PAO1 ΔΔΔ or PAO1 ΔΔΔ Δ*htrB1* Δ*pagP* via the ID (1×10^8 CFU; **B-D**) or IN (2.5×10^7 CFU; **E-G**) route. Four weeks after immunization, half of the mice in each group (n = 8) were challenged IT with DK1-NH57388A embedded in agar beads (1×10^6 CFU). Control mice received sterile beads. Spleens (**B, E**) and lungs (**C, D, F, G**) were collected 24 h before (**B, C, E, F**) and 13 days after challenge (**D, G**). IFN-γ levels in splenocyte and lung cell cultures were measured by ELISA after 3-day stimulation with heat-killed (Hk) PAO1 (orange symbols) or Hk DK1-NH57388A (blue) at a 1:1 T cell to bacterium ratio. Unstimulated lung cells served as controls. Dots represent individual mice; horizontal lines indicate means; error bars, SD. Overall group differences were assessed using Kruskal-Wallis test, followed by Dunn's *post hoc* multiple comparisons test (*p < 0.05, **p < 0.01, ***p < 0.001). S; saline; V1, PAO1 ΔΔΔ; V2, PAO1 ΔΔΔ Δ*htrB1* Δ*pagP*; B, sterile beads; DK1, DK1-NH57388A.

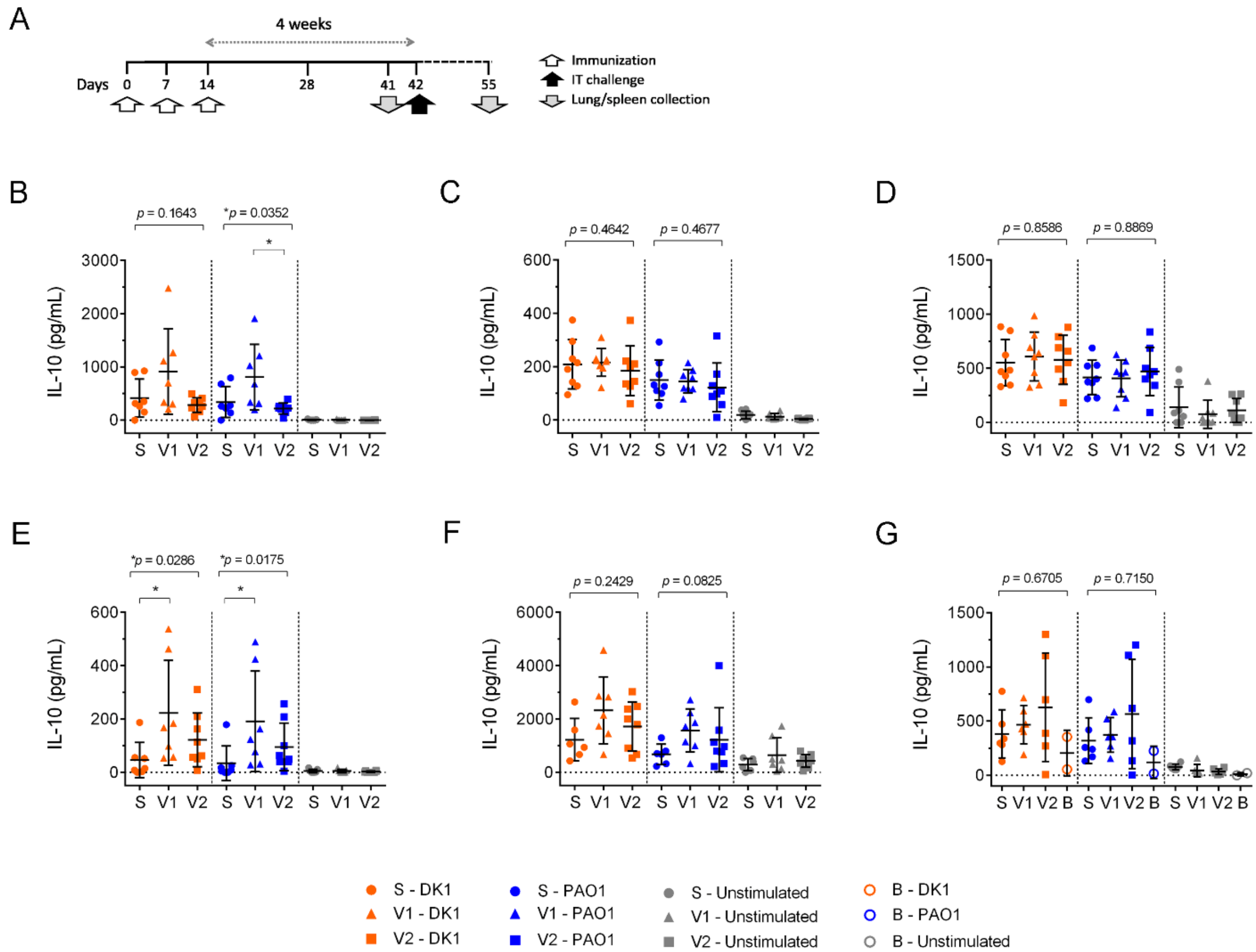

Supplementary Figure 13. IL-10 secretion by $CD4^+$ T cells from spleens and lungs of vaccinated mice. (**A**) Three-dose immunization schedule, challenge, and organ collection. (**B-G**) Mice (n = 16) were immunized with PAO1 ΔΔΔ or PAO1 ΔΔΔ Δ*htrB1* Δ*pagP* via the ID (1×10^8 CFU; **B-D**) or IN (2.5×10^7 CFU; **E-G**) route. Four weeks after immunization, half of the mice in each group (n = 8) were challenged IT with DK1-NH57388A embedded in agar beads (1×10^6 CFU). Control mice received sterile beads. Spleens (**B, E**) and lungs (**C, D, F, G**) were collected 24 h before (**B, C, E, F**) and 13 days after challenge (**D, G**). IL-10 levels in splenocyte and lung cells cultures were measured by ELISA after 3-day stimulation mice with Hk PAO1 (orange symbols) or Hk DK1-NH57388A (blue) at a 1:1 T cell to bacterium ratio. Unstimulated lung cells served as controls. Dots represent individual mice; horizontal lines indicate means; error bars, SD. Overall group differences were assessed using Kruskal-Wallis test, followed by Dunn's *post hoc* multiple comparisons test (*$p < 0.05$). S; saline; V1, PAO1 ΔΔΔ; V2, PAO1 ΔΔΔ Δ*htrB1* Δ*pagP*; B, sterile beads; DK1: DK1-NH57388A.

1.2 Supplementary Tables

Supplementary Table 1. Mortality and lung colonization rates in C57BL/6 mice infected with mucoid *P. aeruginosa* strains isolated from cystic fibrosis patients: strain A (CF-51419182) and strain E (CF-51429475) from CHUAC, strain DK1 (DK1-NH57388A) and the epidemic strain LES400.

Assay	C57BL/6				Infection with *P. aeruginosa* in agar beads		Procedure - Mortality	Acute infection – Mortality	Chronic infection	Lung colonization		
	n	Sex	Age (weeks)	Weight (g ± SD)	Strain	CFU (50 µL)	No. deaths/No. total (%)	No. deaths/No. infected (%)	Time (days)	No. positives /No. total (%)	Lung (g ± SD)	Log_{10} CFU /lung ± SD
#1	15	Male	14	24.65 ± 1.625	A	1.21×10^6	2/15 (13.3)	2/13 (15.4)	16	9/11 (81.8)	0.28 ± 0.038	4.37 ± 0.959
#2	14	Male	11	23.70 ± 2.022	A	5.60×10^5	1/14 (7.1)	0/13 (0)	11	11/13 (84.6)	0.23 ± 0.015	4.54 ± 1.199
#3	15	Male	11	23.17 ± 2.182	DK1	5.50×10^5	3/15 (20)	0/12 (0)	11	10/12 (83.3)	0.21 ± 0.015	4.66 ± 0.718
#4	16	Male	12	22.89 ± 2.179	A	1.65×10^6	1/16 (6.3)	2/15 (13.3)	11	13/13 (100)	0.28 ± 0.053	5.47 ± 0.546
#5	21	Female	10-15	17.95 ± 1.293	A	1.65×10^6	2/21 (9.5)	5/19 (26.3)	11	14/14 (100)	0.26 ± 0.059	5.63 ± 0.440
#6	16	Female	10-12	17.51 ± 0.912	E	5.00×10^5	1/16 (6.3)	0/15 (0)	11	4/15 (26.7)	0.20 ± 0.020	1.77 ± 1.841
#7	14	Male	11-14	23.96 ± 1.887	LES400	1.35×10^6	0/14 (0)	0/14 (0)	11	11/14 (78.6)	0.21 ± 0.012	4.52 ± 2.341
#8	15	Female	12-15	18.87 ± 1.550	DK1	1.64×10^6	1/15 (6.7)	3/14 (21.4)	11	10/11 (90.9)	0.21 ± 0.021	4.77 ± 1.468
#9	14	Male	10-12	23.71 ± 1.468	DK1	1.64×10^6	0/14 (0)	4/14 (28.6)	11	9/10 (90)	0.22 ± 0.038	4.28 ± 0.883
#10	14	Male	8-13	24.03 ± 1.390	DK1	1.98×10^6	0/14 (0)	0/14 (0)	13	10/14 (71.4)	0.23 ± 0.031	5.17 ± 0.608

Supplementary Table 2. Cytokine-producing splenic $CD4^{+}$ T cell percentages in mice vaccinated via intradermal or intranasal route, assessed before and after challenge. Data are shown as mean ± SD. *p*-values for overall group differences (ANOVA) and for pairwise comparisons using Tukey's multiple comparisons test are provided.

Cytokine	Stimulation	Saline		Vaccinated V1		Vaccinated V2		ANOVA	ANOVA with Tukey's multiple comparisons test (*p*-value)		
		n	(%Mean ± SD)	n	(%Mean ± SD)	n	(%Mean ± SD)	*p*-value	S vs. V1	S vs. V2	V1 vs. V2
**Intradermal immunization - Before challenge**											
**IFN-γ**	Non-specfic (PMA/ionomycin)	7	3.18 ± 1.950	8	2.93 ± 1.266	8	1.73 ± 1.030	0.1327	0.9374	0.1512	0.2440
	Specific (hk-PAO1 strain)	–	–	–	–	–	–	–	–	–	–
**IL-4**	Non-specfic (PMA/ionomycin)	7	0.81 ± 0.224	8	0.84 ± 0.270	8	0.70 ± 0.242	0.4677	0.9712	0.6329	0.4709
	Specific (hk-PAO1 strain)	–	–	–	–	–	–	–	–	–	–
**IL-10**	Non-specfic (PMA/ionomycin)	7	0.02 ± 0.007	8	0.02 ± 0.009	8	0.01 ± 0.006	**0.0275 ***	0.6590	**0.0260 ***	0.1304
	Specific (hk-PAO1 strain)	–	–	–	–	–	–	–	–	–	–
**IL-17**	Non-specfic (PMA/ionomycin)	7	0.13 ± 0.061	8	0.16 ± 0.064	8	0.15 ± 0.128	0.8017	0.7922	0.8881	0.9792
	Specific (hk-PAO1 strain)	–	–	–	–	–	–	–	–	–	–
**Intradermal immunization - After challenge**											
**IFN-γ**	Non-specfic (PMA/ionomycin)	7	0.64 ± 0.453	8	1.02 ± 0.274	8	0.85 ± 0.267	0.1242	0.1043	0.4848	0.5735
	Specific (hk-PAO1 strain)	7	0.08 ± 0.039	8	0.52 ± 0.308	8	0.08 ± 0.039	**0.0001 *****	**0.0005 *****	>0.9999	**0.0003 *****
**IL-4**	Non-specfic (PMA/ionomycin)	7	0.42 ± 0.122	8	0.32 ± 0.114	8	0.39 ± 0.095	0.2342	0.2192	0.8148	0.4924
	Specific (hk-PAO1 strain)	7	0.27 ± 0.075	8	0.26 ± 0.071	8	0.34 ± 0.133	0.1917	0.9851	0.3080	0.2178
**IL-10**	Non-specfic (PMA/ionomycin)	7	0.22 ± 0.130	8	0.13 ± 0.041	8	0.13 ± 0.057	**0.0312 ***	**0.0496 ***	0.0524	0.9996
	Specific (hk-PAO1 strain)	7	0.18 ± 0.041	8	0.27 ± 0.133	8	0.17 ± 0.097	0.0970	0.1885	0.9767	0.1155
**IL-17**	Non-specfic (PMA/ionomycin)	7	0.13 ± 0.052	8	0.12 ± 0.042	8	0.10 ± 0.054	0.4561	0.8492	0.4284	0.7388
	Specific (hk-PAO1 strain)	7	0.11 ± 0.009	8	0.29 ± 0.215	8	0.10 ± 0.049	**0.0127 ***	**0.0337 ***	0.9890	**0.0201 ***
**Intranasal immunization - Before challenge**											
**IFN-γ**	Non-specfic (PMA/ionomycin)	6	2.50 ± 2.231	7	0.61 ± 0.240	8	0.63 ± 0.241	**0.0153 ***	**0.0275 ***	**0.0246 ***	0.9994
	Specific (hk-PAO1 strain)	6	0.04 ± 0.048	7	0.06 ± 0.024	8	0.05 ± 0.053	0.7682	0.7966	0.9970	0.8123
**IL-4**	Non-specfic (PMA/ionomycin)	6	2.00 ± 1.934	7	1.19 ± 0.231	8	1.31 ± 0.851	0.4150	0.4329	0.5158	0.9799
	Specific (hk-PAO1 strain)	6	2.70 ± 1.674	7	3.32 ± 2.384	8	0.39 ± 0.481	**0.0075 ****	0.7849	**0.0477 ***	**0.0084 ****
**IL-10**	Non-specfic (PMA/ionomycin)	6	0.09 ± 0.081	7	0.04 ± 0.030	8	0.22 ± 0.553	0.5851	0.9729	0.7488	0.5848
	Specific (hk-PAO1 strain)	6	0.22 ± 0.505	7	0.02 ± 0.033	8	0.04 ± 0.030	0.3560	0.3888	0.4367	0.9898
**IL-17**	Non-specfic (PMA/ionomycin)	6	0.13 ± 0.243	7	0.48 ± 0.300	8	0.60 ± 0.485	0.0866	0.2418	0.0777	0.8076
	Specific (hk-PAO1 strain)	6	1.21 ± 1.087	7	0.79 ± 0.736	8	0.38 ± 0.524	0.1749	0.6064	0.1520	0.5850
**Intranasal immunization - After challenge**											
**IFN-γ**	Non-specfic (PMA/ionomycin)	8	0.96 ± 0.394	8	0.96 ± 0.305	7	1.49 ± 0.545	**0.0392 ***	>0.9999	0.0614	0.0628
	Specific (hk-PAO1 strain)	8	0.36 ± 0.211	8	0.22 ± 0.114	7	0.35 ± 0.089	0.1567	0.1824	0.9854	0.2629
**IL-4**	Non-specfic (PMA/ionomycin)	8	1.64 ± 1.486	8	1.38 ± 0.393	7	2.51 ± 0.792	0.1048	0.8657	0.2411	0.1019
	Specific (hk-PAO1 strain)	8	1.78 ± 0.736	8	1.85 ± 0.617	7	1.84 ± 1.232	0.9853	0.9867	0.9893	>0.9999
**IL-10**	Non-specfic (PMA/ionomycin)	8	0.32 ± 0.160	8	0.26 ± 0.093	7	0.66 ± 0.346	**0.0046 ****	0.8543	**0.0179 ***	**0.0056 ****
	Specific (hk-PAO1 strain)	8	1.32 ± 1.154	8	0.65 ± 0.350	7	1.32 ± 0.469	0.1610	0.2118	>0.9999	0.2311
**IL-17**	Non-specfic (PMA/ionomycin)	8	0.10 ± 0.038	8	0.09 ± 0.038	7	0.18 ± 0.086	**0.0146 ***	0.8618	**0.0479 ***	**0.0165 ***
	Specific (hk-PAO1 strain)	8	0.44 ± 0.285	8	0.19 ± 0.095	7	0.29 ± 0.140	0.0523	**0.0432 ***	0.2990	0.5952
